# How a “Something Else” Response Option for Sexual Identity Affects National Survey Estimates of Associations Between Sexual Identity, Reproductive Health, and Substance Use

**DOI:** 10.1007/s10508-023-02710-7

**Published:** 2023-10-18

**Authors:** Brady T. West, Curtiss W. Engstrom, Ty S. Schepis, Ilmul J. Tani, Sean Esteban McCabe

**Affiliations:** 1 Survey Research Center, 4118 Institute for Social Research, University of Michigan-Ann Arbor, 426 Thompson St., Ann Arbor, MI 48106 USA; 2 Program in Survey and Data Science, University of Michigan-Ann Arbor, Ann Arbor, MI USA; 3 Department of Psychology, Texas State University, San Marcos, TX USA; 4 School of Nursing, University of Michigan-Ann Arbor, Ann Arbor, MI USA

**Keywords:** Sexual orientation, General population cohort studies, Substance use, Reproductive health, Survey measurement

## Abstract

This randomized experiment tested whether the inclusion of a “something else” response option for a question about sexual identity in a national health survey would significantly moderate estimated differences between sexual identity subgroups in terms of various health outcomes, including substance use and reproductive health. We conducted secondary analyses of data from five consecutive years of the National Survey of Family Growth (NSFG; 2015–2019), where two large national half-samples were randomly assigned to receive one of two different versions of a question about sexual identity (a four-category version that included a “something else” response option or a three-category version omitting this option). We focused on national estimates of differences between subgroups defined by sexual identity. Multivariable models indicated that the estimated subgroup differences changed in a statistically significant fashion when using the four-category version of the sexual identity question for several measures, including 16% of male measures (household size, past-year cigarette use, and past-year illicit drug use) and 15% of female measures (wanting a/another child, ever had a sexually transmitted disease, and past-year marijuana use). The absence of a “something else” response option for questions about sexual identity in national health surveys may cause respondents to select options that do not accurately describe their identities, and this can have a significant effect on national estimates of differences between sexual identity subgroups in terms of selected health outcomes.

**Supplementary Information:**

The online version contains supplementary material available at 10.1007/s10508-023-02710-7.

## Introduction

A large body of research has presented evidence of significant differences in the prevalence of adverse health outcomes among subgroups of individuals defined by sexual identity (NASEM, 2020). Individuals who identify as sexual minorities (e.g., lesbian, gay, bisexual) have repeatedly been shown to be at higher risk of substance use disorders (Boyd et al., 2019; Klare et al., 2021; McCabe et al., 2009), psychopathology (e.g., mood disorders) (Bostwick et al., 2010; Denney et al., 2021; Garbarski, 2021), suicidality (Denney et al., 2021; Haney, 2021), and risky sexual behaviors (Charlton et al., 2021; Gurnik et al., 2023; Macapagal et al., 2021). Sexual minorities have also been shown to be at higher risk of discrimination (Casey et al., 2019; Denney et al., 2021; McCabe et al., 2010). Substance misuse is the leading cause of preventable disease and death in the U.S., and sexual minorities are at higher risk for substance-related health consequences (Cochran et al., 2013; HHS, 2020a, b; Veliz et al., 2019). Potential variance in the magnitude of these differences across the lifespan has also been examined (McCabe et al., 2018), suggesting that the significance of this public health problem may vary by age. This growing body of research has led to the implementation of public health policies designed to understand and reduce these differences among sexual identity subgroups at the local, state, and national levels (NASEM, 2020; Tran et al., 2019).

Much of this research has utilized secondary analyses of large national survey data sets that collect measures of both sexual identity and various health outcomes, including the National Survey of Family Growth (NSFG), the National Epidemiologic Survey on Alcohol and Related Conditions (NESARC), and the National Survey of Drug Use and Health (NSDUH). The validity of the estimated differences reported in these studies relies heavily on high-quality measurement of sexual identity, which is critical for the translation of these findings into effective public health policies and programs. Unfortunately, Table 1 below indicates substantial variability across numerous major national health and social surveys in terms of how sexual identity is measured. The effects of these different measurement approaches on population estimates of differences in various health and family formation outcomes among subgroups defined by sexual identity have largely been unexplored to date.

**Table 1:** Variability in measurement of sexual identity across selected major national health surveys

Survey	Year(s)	Sexual identity question wording	Response options
Behavioral Risk Factor Surveillance System (BRFSS)	2018-present	Which of the following best represents how you think of yourself?	Gay/Lesbian or Gay Straight, that is not gay Bisexual Something Else
National Adult Tobacco Survey (NATS)	2013–2014	Do you think of yourself as…?	Gay/Lesbian or Gay Straight, that is not lesbian or gay Bisexual Something Else
National Epidemiologic Survey on Alcohol and Related Conditions-III (NESARC-III)	2012–2013	Which of the categories on the card best describes you?	Heterosexual (straight) Gay or lesbian Bisexual Not sure
National Health Interview Survey (NHIS)	2013-present	Do you think of yourself as gay/lesbian; straight, that is, not gay/lesbian; bisexual; something else; or you don’t know the answer?	Gay/lesbian or gay Straight, that is not lesbian or gay Bisexual Something Else I don't know the answer
National Health and Nutrition Examination Survey (NHANES)	2015-present	Do you think of yourself as lesbian or gay; straight, that is, not lesbian or gay; bisexual; something else; or you don’t know the answer?	Gay/lesbian or gay Straight, that is not lesbian or gay Bisexual Something else I don't know the answer
National Survey on Drug use and Health (NSDUH)	2015-present	Which one of the following do you consider yourself to be?	Heterosexual, that is, straight Lesbian or gay Bisexual
Population Assessment of Tobacco and Health (PATH)	2013-present	Do you think of yourself as…?	Gay/lesbian or gay Straight, that is not gay Bisexual Something Else
National Longitudinal Study of Adolescent to Adult Health (Add Health)	2017–2018	Please choose the description that best fits how you think about yourself	100% Heterosexual (straight) Mostly heterosexual (straight), but somewhat attracted to people of your own sex Bisexual that is, attracted to men and women equally Mostly homosexual (gay), but somewhat attracted to people of the opposite sex 100% homosexual (gay) Not sexually attracted to either males or females
Midlife Development in the U.S. (MIDUS)	2013–2014	How would you describe your sexual orientation? Would you say you are primarily heterosexual (sexually attracted only to the opposite sex), homosexual (sexually attracted only to your own sex), or bisexual (sexually attracted to both men and women)?	Heterosexual Homosexual Bisexual
Household Pulse Survey	2020-present	Which of the following best represents how you think of yourself?	Gay or lesbian Straight, that is not gay or lesbian Bisexual Something else, please specify I don't know
National Crime Victimization Survey (NCVS)	2016-present	Which of the following best represents how you think of yourself?	Gay/lesbian or gay Straight, that is not lesbian or gay Bisexual Something Else I don't know the answer
High School Longitudinal Study of 2009	2009–2018	Do you think of yourself as…	Lesbian or gay, that is, homosexual Straight, that is, heterosexual Bisexual Asexual Something else (please specify) Don't know
California Health Interview Survey (CHIS)	2022	Do you think of yourself as…	{Lesbian or} gay Straight, not {lesbian or} gay Bisexual Something else (Specify) I don't know
Gallup	2012-present	Which of the following do you consider yourself to be? You can select as many as apply	Straight or heterosexual Gay Lesbian Bisexual Transgender
General Social Survey (GSS)	2008-present	Which of the following best describes you?	Gay, lesbian, or homosexual Bisexual Heterosexual or straight
American National Elections Study (ANES)	2008-present	Do you consider yourself to be heterosexual or straight, homosexual or gay (or lesbian), or bisexual?	Heterosexual or straight Homosexual or gay (or lesbian) Bisexual
Youth Risk Behavior Surveillance System (YRBSS)	2015-present	Which of the following best describes you?	Heterosexual (straight) Gay or lesbian Bisexual Not sure
LGBT Survey (EU)	2012	PLEASE SELECT THE ONE ANSWER THAT FITS YOU BEST	Lesbian Gay Bisexual Heterosexual/straight Other, please write:
Health Information National Trends Survey (HINTS)	2017-present	Do you think of yourself as…	Heterosexual or straight Homosexual or gay (or lesbian) Bisexual Something else
Labour Force Survey (LFS; UK)	2012-Present	Which of the following best describes your sexual orientation?	Straight or heterosexual Gay or lesbian Bisexual Other
Survey of Safety in Public and Private Spaces (Canada)	2018-Present	What is your sexual orientation?	Heterosexual Homosexual (e.g., lesbian or gay) Bisexual Please specify
Scottish Household Survey (Scotland)	2011-present	Which of the following options best describes how you think of yourself?	Heterosexual/straight Gay/lesbian Bisexual Other

The survey methodology field is well-aware of this critical measurement issue (Eliason et al., 2016; FSCM, 2016a; Ridolfo et al., 2012). An entire 2019 issue of the *Journal of Official Statistics* was dedicated to research on the survey measurement of sexual identity (Volume 35, Issue 4: *Special Issue on Measuring LGBT Populations*). The Federal Committee on Statistical Methodology recently established an inter-agency interest group dedicated to research on the measurement of sexual orientation and gender identity (https://nces.ed.gov/FCSM/interagency_reports.asp). Based in part on the work of this interest group, the Office of the Chief Statistician of the United States recently released a report providing recommended best practices for collecting sexual orientation and gender identity data in Federal surveys (Office of the Chief Statistician of the United States, 2023). The National Academies of Sciences, Engineering, and Medicine (NASEM) also recently released a report describing an extensive inter-agency study of best practices for measuring sexual orientation and gender identity (NASEM, 2022). Unfortunately, many of the national surveys used to generate this influential body of research on health differences related to sexual identity were fielded prior to these more recent attempts to establish best practices. These surveys generally collected relatively simple closed-ended measures about sexual identity that required respondents to choose from a limited number of response options (i.e., straight, lesbian/gay, or bisexual, with no other choices provided) (FCSM, 2016b).

Using sexual identity questions with limited response options introduces a risk of survey respondents being misclassified in terms of their sexual identity, especially if respondents do not perceive that the options provided apply to them (Dragan & Folkierska-Żukowska, 2022; see also the discussion in Everett, 2013). The minority stress model (Meyer, 2003, 2015) is one theoretical model proposing that members of sexual minority subgroups experience greater stigma and stressors than do heterosexual individuals. In response to this stress, sexual minority individuals may not outwardly identify as a member of a sexual minority subgroup, instead choosing a less stigmatizing option like heterosexual or “something else”. This misclassification may be heightened in racial/ethnic minority groups, those with high levels of internalized homophobia, or those living in less accepting environments (Amola & Grimmett, 2015; Denison et al., 2021; McConnell et al., 2018).

The statistical literature has clearly established that this type of misclassification in the measures on a categorical variable (like sexual identity) will attenuate estimated associations between that categorical variable and other measures (West & McCabe, 2021). Thus, national estimates of health disparities among sexual identity subgroups may be understated and/or misleading, and recent work has confirmed this possibility (West & McCabe, 2021). This survey measurement issue therefore has critical implications for evidence-based public health policies and practices.

In the current study, we present secondary analyses of data collected from a split-sample experiment embedded in five years of the NSFG (2015–2019), extending preliminary work examining this issue (West & McCabe, 2021). We focus on a variety of health outcomes, including reproductive health and related attitudes, and examine whether the same patterns of attenuated differences between sexual identity subgroups reported in previous work on substance use emerge for these additional health measures (West & McCabe, 2021). Based on this prior work and the theoretical mechanisms outlined above, we hypothesize that estimated differences between the sexual identity subgroups in terms of these additional health measures will be attenuated when using a smaller number of response options for sexual identity. With this work, we hope to motivate the continued development and use of improved measures of sexual identity for studies aiming to compare health outcomes among sexual identity subgroups.

## Method

### National Survey of Family Growth Overview

The NSFG collects fertility and family formation data in 60–80 min in-person interviews conducted with a national area probability sample of individuals aged 15 to 49. The NSFG sample design features stratified multistage cluster sampling of households and age-eligible individuals within households (Lepkowski et al., 2013). The NSFG data collection is continuous, with national probability samples released every quarter. We analyzed public-use NSFG data collected from a national probability sample of more than 21,441 U.S. males and females between 2015 and 2019. For additional details about the design of the NSFG, including response rates from these years (which generally averaged 70%), see the technical reports for these data prepared by the National Center for Health Statistics (NCHS, 2018; NCHS 2020).

### Sexual Identity Experiment

Between the years of 2015 and 2019, the NSFG implemented a unique split-sample experiment related to the measurement of sexual identity during data collection (the experiment was not implemented in any other years). Half of the 2015–2019 NSFG sample (here forth labelled TG1) was randomly assigned to receive one version of the sexual identity question during audio computer-assisted self-interviewing (ACASI), and half of the sample was randomly assigned to receive the other version (HHS, 2018; NSFG, 2021). The first “three-category” version (TG1) was unchanged from previous NSFG years: “Do you think of yourself as…heterosexual or straight (1), homosexual or gay/lesbian (2), or bisexual (3)?” The second “four-category” version (here forth labelled TG2) was drawn from the PhenX Toolkit: “Which of the following best represents how you think of yourself? Lesbian/gay (1), straight, that is, not lesbian/gay (2), bisexual (3), or something else (4)?” Item non-response rates for these two sexual identity questions were small (1.5% of women and 1.0% of men for TG1, and 1.0% of women and 0.8% of men for TG2) and did not vary significantly between the half-samples (West & McCabe, 2021).

In theory, adding the fourth response option of “Something Else” helps to improve the measurement of sexual identity. A recent NASEM report (2020) called for “…methodological research to develop, improve, and expand measures that capture the full range of sexual and gender diversity in the population—including but not limited to intersex status and emerging sexual and gender identities…” Write-in options for “something else” that would enable more evaluation of these emerging sexual identities were not collected for TG2, so we cannot qualitatively examine the responses of these individuals.

While the wording of these two questions in TG1 and TG2 was quite similar, the TG2 version (Dahlhamer et al., 2014) did not use the terms “heterosexual” or “homosexual” in the response options because some respondents find these terms confusing (Ridolfo et al., 2012). We also note that the ordering of the responses varied in the two questions. Response-order effects are generally more prominent in telephone surveys, where respondents cannot see the survey questions and response options (Holbrook et al., 2003). Self-administration of these types of sensitive questions does not introduce substantial effects of response ordering (Bishop et al., 1988; Sykes and Collins, 1988). Primacy effects previously reported for “speeders” and respondents with lower education in web surveys with no interviewer present would also likely be mitigated by the interviewer presence during ACASI in the NSFG (Galesic et al., 2008; Malhotra, 2008). We therefore have no theoretical reason to expect that the different ordering of the response options in the two versions of this question would affect our analyses.

Finally, the TG2 version also contains the qualifier “that is, not lesbian/gay” in the “Straight” category. Ridolfo and colleagues (2012) noted that this is important, as it allows respondents to identify with “not-me identities,” constructed through a process of dis-identification with an often-stigmatized group. This subtle difference may have resulted in slightly different populations identifying with the “Straight” category across TG1 and TG2; we will examine this possibility as part of our analytic approach.

### Survey Measures

We computed estimates of sexual identity subgroup differences in the distributions of selected measures that met the following criteria:

1. They are of high scientific interest to researchers studying reproductive health and family formation (see https://www.cdc.gov/nchs/nsfg/nsfg_products.htm) and
2. They have been the focus of prior studies where sexual identity and possibly interactions involving sexual identity were predictors of the measure (see the studies cited in the list of measures below).

These measures (with possible values in parentheses) included:

1. Current marital status (married vs. not married), along with number of times married (0, 1, 2,…; Kerridge et al., 2017);
2. Family formation, including household size (1, 2, 3, …) and an indicator of currently living with at least one child under the age of 18 (yes/no; Weber, 2008);
3. Current pregnancy status (yes/no; Charlton et al., 2020; Everett et al., 2017, 2019a);
4. Current use of various types of contraceptives (e.g., condoms; yes/no, for each type; Charlton et al., 2013, 2019);
5. Current sexual activity without contraceptive use (yes/no, for those sexually active; Charlton et al., 2013, 2019);
6. Intention to have children in the future (yes/no; Shenkman & Abramovitch, 2021);
7. Measures of current substance use, including past-month binge drinking; past-year cigarette smoking, including at the rate of a pack-per-day; marijuana use; and other illicit drug use (e.g., cocaine, crack, and crystal meth) (yes/no; Boyd et al., 2019; Drabble et al., 2021; Klare et al., 2021);
8. Risky sexual behaviors, including number of current sex partners (0, 1, 2,…) and anal sex (ever/never; Parmenter et al., 2020; Ueda et al., 2020); and
9. Measures of sexual health, including sexually transmitted diseases (ever had an STD/never had an STD, and past-year STD (yes/no); Gurnik et al., 2023; Everett et al., 2019a, 2019b).

Additional covariates considered included age in years, race (white, black, other), education (less than high school (HS), HS, greater than HS), and total family income ($0–$19,999, $20K–$34,999, $35K-$69,999, $70K+). As the concept of “straight” does not resonate culturally with Spanish speakers (Ridolfo et al., 2012), we also analyzed an indicator of Hispanic ethnicity (yes, no).

### Statistical Analysis

We used the NSFG survey weights to compute design-unbiased estimates of population parameters and account for the complex sampling features of the NSFG when estimating standard errors and testing hypotheses. All bivariate tests of associations between sexual identity and categorical health measures employ design-adjusted Rao-Scott tests. We used multivariable models to compute adjusted estimates of subgroup differences in the distributions of the health outcomes; we fit both linear and logistic regression models using the pseudo-maximum likelihood estimation method for complex samples, and use design-adjusted subpopulation analyses and goodness of fit assessments (e.g., area under the ROC curve, or AUC) when appropriate (Heeringa et al., 2017). All analyses are stratified by sex, given evidence of larger increases in the risk of adverse health outcomes for sexual minority women (Boyd et al., 2019; McCabe et al., 2005). All analyses use Stata (version 17) commands for the analysis of complex sample survey data.

We first performed descriptive comparisons of the weighted percentages and means for the various health outcomes for each sexual identity subgroup defined by the two NSFG treatment groups. This enabled comparisons of the estimated associations between sexual identity and the various outcome measures across the two samples, via the Rao-Scott tests and design-adjusted Wald tests (for the means). Next, we fit linear and logistic regression models to predict the various continuous and binary health outcomes with the different measures of sexual identity and the covariates. We formally compared the sexual identity subgroup differences between the two NSFG treatment groups by testing two-way interactions between sexual identity and the treatment group indicator. In the comparative analyses, we dropped respondents indicating “something else” in NSFG TG2, enabling comparisons across the treatment groups of estimated differences in the health outcomes between the more commonly endorsed sexual identities (straight or heterosexual, gay (for males) or lesbian (for females), and bisexual).

Following recommendations from Rothman (1990) and Gardner and Altman (1986), we focused primarily on effect sizes associated with the two-way interactions of interest in these models (and their confidence intervals). We examined the estimated interaction coefficients in these models, along with their design-adjusted 95% confidence intervals, in addition to design-adjusted Wald tests for each two-way interaction, using a 0.05 level of significance, and used these criteria to identify outcomes where there was evidence of substantial (i.e., nonzero) moderation of the subgroup differences based on the measurement approach. For these outcomes, we computed estimates of odds ratios quantifying differences between the sexual identity subgroups (and their 95% confidence intervals) based on the estimated coefficients in the models. We then used the margins and marginsplot post-estimation commands in Stata to compute and visualize marginal predictions of the subgroup differences for each measurement approach (adjusting for the covariates in the models), along with design-adjusted 95% confidence intervals for the differences. This approach enabled visualization of any estimated subgroup differences that varied substantially depending on the measurement approach.

Given rates of item-missing data on the measures of interest that varied between 0.2 and 2.3% (for females) and 0.5 and 2.9% (for males), the sample sizes used to fit the multivariable models varied depending on the measure. We also performed a sensitivity analysis and repeated all analyses described above after conducting a multiple imputation analysis. We generated multiple (10) imputations of each missing value using a chained equations approach (Raghunathan et al., 2001), where we first imputed modal values for measures with less than 100 missing values, and then employed chained equations depending on the type of each measure. We imputed the four possible categories of sexual identity for both treatment groups. We recoded imputed values of “something else” in TG1 by first generating a random draw from a Uniform(0,1) distribution, and imputing one of the three response categories for TG1 by referring the random draw to the marginal distribution of sexual identity for TG1 based on complete cases (i.e., if the random draw was between 0 and 0.883 for females, per Table 2, the “something else” respondent was imputed to be heterosexual, and if the random draw was between 0.884 and 0.910, they were imputed to be lesbian). Estimates and their standard errors based on each imputed data set were combined using the combining rules described in Little and Rubin (2019).

**Table 2:** Estimated prevalence of sexual identity subgroups by treatment groups (NSFG 2015–2019)

Sexual identity response options	Women		Men	
	TG1	TG2	TG1	TG2
	% (*n*)	% (*n*)	% (*n*)	% (*n*)
Heterosexual/straight	88.3 (4697)	86.0 (4362)	94.5 (4085)	91.8 (3861)
Gay/lesbian	2.7 (144)	2.0 (120)	2.5 (113)	2.9 (132)
Bisexual	7.6 (459)	7.4 (409)	2.1 (111)	2.2 (105)
Something else	–	3.6 (201)	–	2.3 (103)
Don’t know	0.6 (23)	0.2 (6)	0.2 (14)	0.1 (4)
Refused	0.9 (56)	0.8 (47)	0.8 (46)	0.7 (29)
Sample size	5379	5145	4369	4234

## Results

### Estimated Sexual Identity Distributions

Table 2 below presents unweighted sample sizes and weighted estimates of the sexual identity distributions by sex based on the two sample subgroups (TG1 and TG2) in the 2015–2019 NSFG data (using the final NSFG survey weights).

In Table 2, we note that at least 100 women and men responded with the “something else” option when given the chance, meaning that this sexual identity subgroup represents a non-negligible 2–4% of the larger target population. Relative to the distributions based on TG1, we see that offering the “something else” option reduces the estimated percentages of both men and women who identify as heterosexual/straight, and of women who identify as lesbian or bisexual.

### Comparisons of Associations

Table 3 presents the estimated bivariate associations between sexual identity and selected health outcomes for males, along with the design-adjusted Rao-Scott or Wald tests of the associations, separately for TG1 and TG2.^1^

**Table 3:** Estimated prevalence of substance use, family formation, and sexual behavior outcomes by sexual identity subgroups among men (NSFG 2015–2019)

Treatment group	TG1			TG2			
Sexual identity	Heterosexual/straight	Gay	Bisexual	Heterosexual/straight	Gay	Bisexual	Something Else
	% (95% CI) *n*	% (95% CI) *n*	% (95% CI) *n*	% (95% CI) *n*	% (95% CI) *n*	% (95% CI) *n*	% (95% CI) *n*
**Past-Month Binge Drinking**	44.0% (41.4, 46.6) *n* = 4061	46.2% (35.6, 57.1) *n* = 113	40.8% (28.5, 54.5) *n* = 110	44.2% (41.5, 46.9) *n* = 3844	46.4% (32.3, 61.1) *n* = 131	35.4% (24.4, 48.1) *n* = 105	60.9% (46.8, 73.4) *n* = 105
Rao-Scott Adjusted *F*-Test	*F*(1.9, 205.3) = 0.2, *p* = .8			*F*(1.9, 200.7) = 0.7, *p* = .5			
**Past Year Pack-a-Day Smoker**	9.0% (7.6, 10.6) *n* = 4083	7.5% (3.5, 15.4) *n* = 113	10.3% (4.7, 21.1) *n* = 111	7.3% (6.2, 8.6) *n* = 3858	4.0% (1.8, 8.8) *n* = 132	5.3% (2.3, 11.9) *n* = 105	6.7% (2.5, 16.6) *n* = 103
Rao-Scott Adjusted *F*-Test	*F*(2.0, 211.3) = 0.2, *p* = .8			*F*(2.0, 213.6) = 1.3, *p* = .3			
**Past-Year Cigarette Use**	24.5% (22.2, 26.9) *n* = 4,083	20.3% (12.1, 31.9) *n* = 113	29.8% (19.9, 42.2) *n* = 111	23.8% (21.7, 26.1) *n* = 3,858	27.7% (17.5, 40.9) *n* = 132	14.6% (9.2, 22.5) *n* = 105	30.9% (18.6, 46.6) *n* = 103
Rao-Scott Adjusted *F*-Test	*F*(2.0, 215.5) = 0.8, *p* = .5			*F*(1.6, 169.5) = 1.8, *p* = .2			
**Past-Year Marijuana Use**	28.4% (26.2, 30.7) *n* = 4076	50.8% (38.2, 63.2) *n* = 112	44.6% (32.4, 57.4) *n* = 111	27.5% (25.5, 29.5) *n* = 3835	45.8% (34.4, 57.6) *n* = 132	39.6% (27.7, 52.8) *n* = 103	51.3% (39.1, 63.4) *n* = 103
Rao-Scott Adjusted *F*-Test	*F*(2.0, 210.9) = 11.1, *p* < .001			*F*(1.9, 207.9) = 8.0, *p* < .001			
**Past-Year Other Drug Use**^**a**^	5.3% (4.5, 6.3) *n* = 4,083	12.0% (6.2, 21.8) *n* = 113	18.7% (10.8, 30.3) *n* = 111	5.9% (4.9, 7.1) *n* = 3,855	6.8% (3.5, 12.7) *n* = 132	6.3% (3.0, 12.5) *n* = 104	13.8% (7.6, 23.9) *n* = 103
Rao-Scott Adjusted *F*-Test	*F*(2.0, 215.7) = 12.9, *p* < .001			*F*(2.0, 211.6) = 0.1, *p* = .9			
**Married?**	45.1% (42.4, 47.8) *n* = 4,085	0% *n* = 111	20.5% (12.0, 32.7) *n* = 111	43.3% (40.3, 46.4) *n* = 3,859	9.6% (3.0, 26.9) *n* = 130	21.1% (12.7, 33.1) *n* = 105	30.1% (19.3, 43.7) *n* = 103
Rao-Scott Adjusted *F*-Test	*F*(2.0, 212.2) = 29.0, *p* < .001			*F*(1.6, 174.9) = 12.6, *p* < .001			
**Number of Times Married (Mean)**	0.6 (0.6, 0.7) *n* = 4,085	0.1 (− 0.0, 1.4) *n* = 113	0.3 (0.2, 0.4) *n* = 111	0.6 (0.6, 0.7) *n* = 3861	0.2 (− 0.0, 0.3) *n* = 132	0.4 (0.2, 0.6) *n* = 105	0.5 (0.3, 0.7) *n* = 103
Design-Adjusted *F*-Test	*F*(2, 107) = 91.6, *p* < .001			*F*(2, 107) = 13.6, *p* < .001			
**Household Size (Mean)**	3.4 (3.3, 3.5) *n* = 4,085	2.6 (2.3, 2.9) *n* = 113	2.9 (2.6, 3.2) *n* = 111	3.3 (3.2, 3.4) *n* = 3,861	2.8 (2.3, 3.2) *n* = 132	3.3 (2.8, 3.7) *n* = 105	3.2 (3.0, 3.4) *n* = 103
Design-Adjusted *F*-Test	*F*(2, 107) = 17.6, *p* < .001			*F*(2, 107) = 1.7, *p* = .2			
**Children Under 18 in HH**	44.0% (41.4, 46.6) *n* = 4085	6.7% (1.9, 21.1) *n* = 113	13.4% (7.3, 23.3) *n* = 111	39.7% (36.5, 43.0) *n* = 3861	8.4% (2.7, 23.4) *n* = 132	18.7% (11.7, 28.4) *n* = 105	39.0% (26.6, 35.0) *n* = 103
Rao-Scott Adjusted *F*-Test	*F*(1.7, 181.4) = 22.5, *p* < .001			*F*(1.5, 166.0) = 14.1, *p* < .001			
**Want a/another Child**	58.9% (56.0, 61.8) *n* = 3972	53.6% (38.3, 68.2) *n* = 109	63.2% (48.2, 76.1) *n* = 104	61.7% (59.1, 64.2) *n* = 3770	41.5% (29.4, 54.8) *n* = 129	67.2% (54.0, 78.2) *n* = 99	68.8% (55.8, 79.4) *n* = 98
Rao-Scott Adjusted *F*-Test	*F*(1.8, 190.3) = 0.4, *p* = .6			*F*(1.5, 166.0) = 14.1, *p* < .001			
**Every Had a Vasectomy?**	7.5% (6.2, 9.0) *n* = 4081	0% *n* = 113	3.1% (1.1, 8.6) *n* = 111	8.0% (6.6, 9.7) *n* = 3858	0% *n* = 132	2.7% (0.7, 9.4) *n* = 105	0.8% (0.1, 5.7) *n* = 103
Rao-Scott Adjusted *F*-Test	*F*(1.6, 169.8) = 3.1, *p* < .1			*F*(1.8, 191.1) = 4.3, *p* < .05			
**Lifetime Sexual Activity Without a Vasectomy**	92.2% (90.6, 93.5) *n* = 3838	100% *n* = 107	96.6% (90.6, 98.8) *n* = 101	91.5% (89.8, 93.0) *n* = 3628	100% *n* = 126	96.8% (88.9, 99.1) *n* = 96	99.2% (94.2, 99.9) *n* = 94
Rao-Scott Adjusted *F*-Test	*F*(1.6, 168.7) = 3.1, *p* < .1			*F*(1.8, 194.0) = 4.3, *p* < .05			
**Condom Use during Last Sex (Currently Sexually Active)**	37.9% (35.6, 40.2) *n* = 3,815	57.0% (44.5, 68.7) *n* = 107	80.9% (68.0, 89.5) *n* = 100	39.4% (36.8, 42.1) *n* = 3,595	66.5% (54.5, 76.8) *n* = 126	66.4% (54.1, 76.9) *n* = 96	45.5% (32.3, 59.3) *n* = 94
Rao-Scott Adjusted *F*-Test	*F*(2.0, 214.0) = 24.2, *p* < .001			*F*(1.9, 208.5) = 19.5, *p* < .001			
**No Vasectomy, Condom Not Used During Last Sexual Activity (Full Sample)**	52.3% (49.8, 54.7) *n* = 4084	42.1% (30.6,54.5) *n* = 113	16.3% (8.8,28.3) *n* = 111	49.8% (47.3,52.4) *n* = 3859	32.5% (22.7,44.1) *n* = 132	27.8% (18.6,39.4) *n* = 105	52.1% (38.8,65.2) *n* = 103
Rao-Scott Adjusted *F*-Test	*F*(2.0, 214.6) = 15.1, *p* < .001			*F*(2.0, 211.4) = 10.3, *p* < .001			
**Number of Lifetime Sexual Partners (Mean)**	4.8 (4.7, 5.0) *n* = 4,001	6.6 (5.9, 7.4) *n* = 109	7.3 (5.9, 7.4) *n* = 109	4.7 (4.5, 4.9) *n* = 3,776	6.5 (5.9, 7.0) *n* = 126	6.2 (4.7, 7.7) *n* = 104	5.2 (4.6, 5.8) *n* = 102
Design-Adjusted *F*-test	*F*(2, 107) = 25.5, *p* < .001			*F*(2, 107) = 17.3, *p* < .001			
**Number of Past-Year Sexual Partners (Mean)**	1.1 (1.1, 1.2) *n* = 4,048	1.7 (1.5, 2.0) *n* = 113	1.6 (1.3, 1.9) *n* = 110	1.1 (1.1, 1.2) *n* = 3,821	1.7 (1.5, 2.0) *n* = 130	1.4 (1.0, 1.7) *n* = 105	1.2 (1.0, 1.4) *n* = 101
Design-Adjusted *F*-test	*F*(2, 107) = 14.5, *p* < .001			*F*(2, 107) = 12.2, *p* < .05			
**Lifetime Report of Anal Sex**	42.8% (40.1, 45.7) *n* = 4081	91.0% (83.3, 95.3) *n* = 113	70.8% (58.1, 80.9) *n* = 111	40.7% (38.2, 43.3) *n* = 3859	89.5% (82.5, 93.9) *n* = 132	65.6% (48.2, 79.7) *n* = 105	48.8% (34.6, 63.3) *n* = 103
Rao-Scott Adjusted *F*-Test	*F*(1.8, 197.5) = 44.6, *p* < .001			*F*(1.7, 185.6) = 37.4, *p* < .001			
**Ever had an STD**	4.2% (3.3, 5.3) *n* = 4078	25.0% (14.9, 38.7) *n* = 113	17.1% (9.4, 29.1) *n* = 110	2.8% (2.2, 3.7) *n* = 3851	18.6% (11.3, 29.1) *n* = 132	6.2% (2.4, 15.0) *n* = 105	9.3% (3.5, 22.7) *n* = 103
Rao-Scott Adjusted *F*-Test	*F*(1.9, 208.1) = 35.2, *p* < .001			*F*(2.0, 213.1) = 30.7, *p* < .001			
**Past-Year Report of an STD**	0.5% (0.3, 0.7) *n* = 4077	6.5% (3.1, 13.1) *n* = 113	3.2% (1.1, 9.3) *n* = 110	1.1% (0.7, 1.7) *n* = 3850	5.4% (1.8, 14.8) *n* = 132	2.5% (1.0, 6.5) *n* = 105	2.1% (0.6, 7.1) *n* = 103
Rao-Scott Adjusted *F*-Test	*F*(2.0, 215.2) = 35.6, *p* < .001			*F*(1.6, 168.5) = 6.8, *p* < .01			

^a^Other drug use includes the use of cocaine, crack, and methamphetamines

In Table 3, the reported tests of associations considered the largest three categories of sexual identity (heterosexual/straight, gay, and bisexual) to see if estimated differences between these categories would be sensitive to the inclusion of the “something else” response option in TG2. Consider the three outcomes measuring past-year cigarette use, past-year other drug use, and wanting a/another child. We see that males who identify as “something else” if given the option (TG2) tend to have a higher prevalence of each of these outcomes, and that estimates of differences between individuals who identify as heterosexual and gay or bisexual tend to change depending in the measurement approach. This is particularly true for other drug use and wanting a/another child, where the Rao-Scott tests are completely different; this is also the case for mean household size. Based on the sample that was only provided with three sexual identity options (TG1), the estimated prevalence of the three binary outcomes for the gay and bisexual subgroups tends to be larger, where presumably the individuals identifying as “something else” in TG2 (who tend to be more likely to endorse each of these outcomes) are forced to choose between the three options provided.

For example, consider other drug use in the past year. When using the four-category measure of sexual identity, the estimated prevalence is 13.8% among those responding with “something else”, compared to only 6.8% for those responding as gay. Table A8 in the online Appendix shows that the “something else” subgroup continues to have a marginally higher prevalence of substance use than the other three groups in a multivariable analysis. The resulting difference in prevalence between gay and bisexual males is very small, and the Rao-Scott test of association (*p* = .9) does not suggest any association of sexual identity with this outcome. However, when considering the estimates based on the three-category measure, we see much larger estimates of differences in prevalence between gay or bisexual males and heterosexual males (*p* < .001); notably, the estimated prevalence for heterosexual males remains quite similar regardless of the measure of sexual identity used. Respondents who might have indicated “something else” if provided with this option may have selected either gay or bisexual instead, increasing those estimated prevalence rates.

When following our multivariable modeling approach to determine which of these changes in associations remained large in magnitude after adjusting for relevant covariates, we identified three outcomes where the models had non-zero two-way interactions between sexual identity and the measurement approach (TG1 vs. TG2), with a design-adjusted Wald test *p* value less than .05 and corresponding interaction coefficients with 95% confidence intervals (CIs) that did not include zero. These included past-year cigarette use, past-year other drug use, and household size. See Table A4 in the online Appendix for the estimated coefficients, 95% CIs, and corresponding design-adjusted Wald tests. Notably, there was no longer evidence of a two-way interaction for wanting a/another child when adjusting for the covariates. This may have been due to shifts in socio-demographic measures related to this outcome that were also engendered by the different measurement approaches; see Table A3a in the online Appendix.

Based on the model for past-year cigarette use, the 95% CIs for the odds ratios comparing gay and bisexual individuals to heterosexual individuals in TG1 both included one, suggesting negligible differences based on TG1. Based on TG2, the estimated odds ratio comparing bisexual individuals to heterosexual individuals was 0.42 (95% CI = 0.22, 0.83), suggesting a non-zero 58% reduction in the odds of past-year cigarette use for bisexuals relative to heterosexuals; the 95% CI for the gay vs. heterosexual odds ratio continued to include 1. Consistent with Table 3, we would arrive at a completely different conclusion about the bisexual vs. heterosexual difference in this behavior depending on the measurement approach.

Based on the model for past-year other drug use, the estimated odds ratios for gay vs. heterosexual (OR = 2.23, 95% CI = 1.03, 4.81) and bisexual vs. heterosexual (OR = 3.56, 95% CI = 1.84, 6.90) in TG1 both had 95% CIs that did not include one, suggesting non-zero differences with increased drug use for sexual minorities (consistent with Table 3). In contrast, the 95% CIs both included one for TG2, suggesting negligible differences. We would again arrive at completely different conclusions about these differences after adjusting for the covariates, depending on the measurement approach. Based on the model for household size, gay and bisexual individuals were both estimated to have substantially lower mean household sizes than heterosexual respondents (estimated coefficients = − 0.75 and − 0.56, with 95% CIs of (− 1.07, − 0.43) and (− 0.83, − 0.29), respectively) in TG1 (again consistent with Table 3). In TG2, these differences disappeared, with both 95% CIs for the differences including zero.

**Fig. 1:**
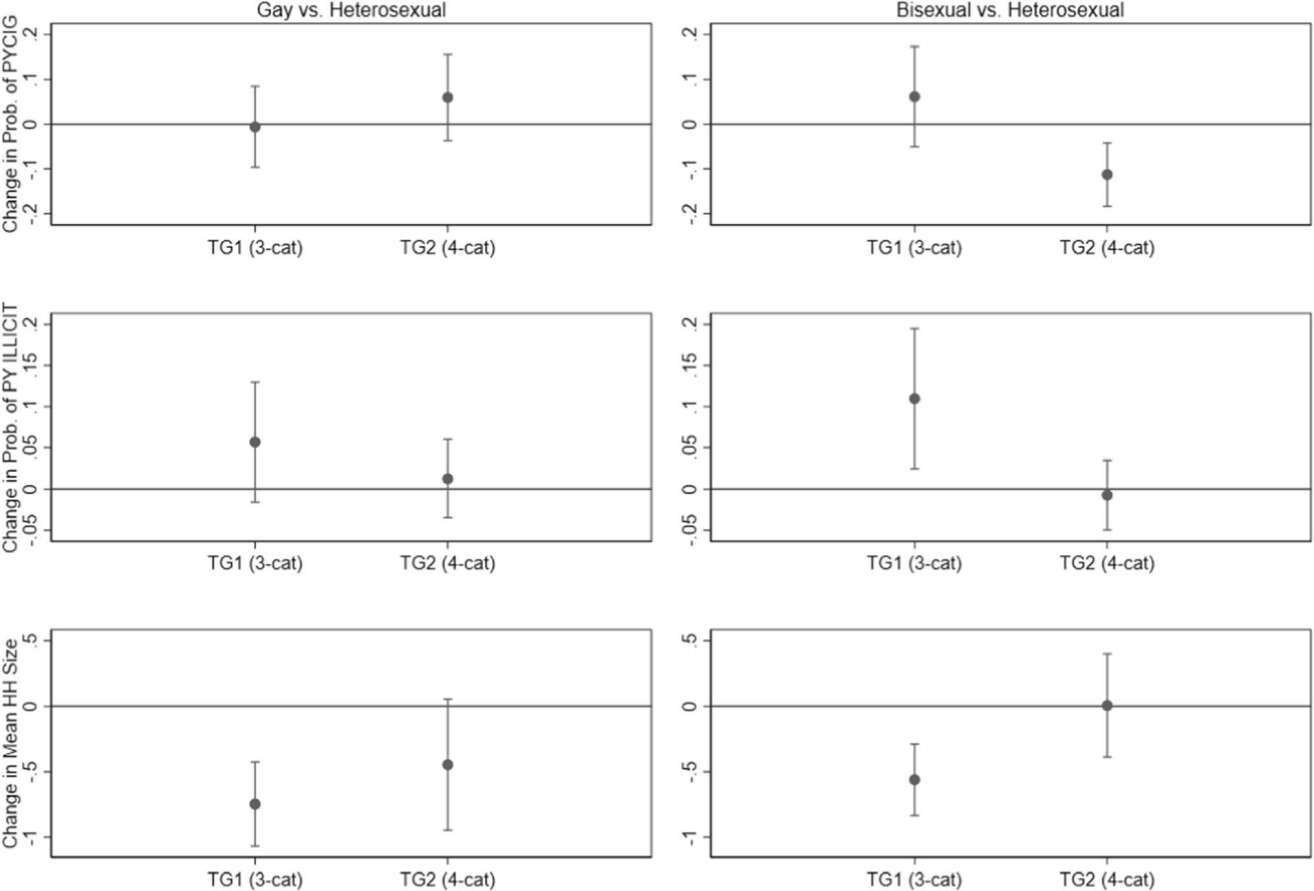
Estimates of marginal differences between gay and heterosexual males (first column) and bisexual and heterosexual males (second column) in the probabilities or means of selected outcomes by measurement approach (TG1 = three categories of sexual identity, TG2 = four categories of sexual identity), including 95% CIs for the marginal differences, based on estimated multivariable models (PYCIG = past-year cigarette use; PYILLICIT = past-year other drug use; HH Size = household size)

Based on these modeling results, Fig. 1 displays estimates of the marginal differences between gay and heterosexual individuals (first column) and between bisexual and heterosexual individuals (second column) in terms of these outcomes (adjusting for the covariates in the models), along with 95% CIs for the marginal differences. Figure 1 provides a clear visualization of these estimated differences depending on the measurement approach, where in one case a 95% CI for the difference may include zero, and in a second case it does not.

Table 4 presents the estimated bivariate associations between sexual identity and selected health outcomes for females, along with the design-adjusted Rao-Scott or Wald tests of the associations, separately for TG1 and TG2.

**Table 4:** Estimated prevalence of substance use, family formation, and sexual behavior outcomes by sexual identity subgroups among women (NSFG 2015–2019)

Treatment Group	TG1			TG2			
Sexual identity	Heterosexual/Straight	Lesbian	Bisexual	Heterosexual/Straight	Lesbian	Bisexual	Something Else
	% (95% CI) *n*	% (95% CI) *n*	% (95% CI) *n*	% (95% CI) *n*	% (95% CI) *n*	% (95% CI) *n*	% (95% CI) *n*
**Past-Month Binge Drinking**	31.9% (29.7, 34.1) *n* = 4,682	46.4% (32.9, 60.4) *n* = 143	43.6% (37.2, 50.3) *n* = 457	31.1% (28.6, 33.6) *n* = 4,353	35.2% (24.5, 47.5) *n* = 120	48.2% (41.3, 55.2) *n* = 409	30.6% (22.4, 40.3) *n* = 200
Rao-Scott Adjusted *F*-Test	*F*(1.9, 200.2) = 7.5, *p* < .01			*F*(2.0, 213.2) = 13.3, *p* < .001			
**Past-Year Cigarette Use**	19.6% (17.5, 21.9) *n* = 4,698	29.3% (20.5, 39.9) *n* = 144	32.1% (26.3, 38.5) *n* = 459	16.8% (14.9, 19.0) *n* = 4,362	31.0% (18.6, 47.1) *n* = 120	38.2% (31.5, 45.4) *n* = 409	14.7% (9.9, 21.2) *n* = 201
Rao-Scott Adjusted *F*-Test	*F*(1.9, 208.6) = 12.0, *p* < .001			*F*(1.9, 210.7) = 24.5, *p* < .001			
**Past Year Pack-a-Day Smoker**	5.0% (3.8, 6.4) *n* = 4,698	6.5% (3.7, 11.2) *n* = 144	7.9% (4.7, 13.1) *n* = 459	5.0% (3.9, 6.5) *n* = 4,362	15.3% (5.9, 34.2) *n* = 120	11.6% (8.1, 16.2) *n* = 409	2.3% (1.0, 5.0) *n* = 201
Rao-Scott Adjusted *F*-Test	*F*(1.7, 180.7) = 2.2, *p* = .124			*F*(1.7, 186.2) = 7.8, *p* < .001			
**Past-Year Marijuana Use**	18.6% (16.9, 20.4) *n* = 4,694	38.1% (29.1, 47.9) *n* = 143	42.9% (36.3, 49.9) *n* = 458	17.8% (15.9, 19.7) *n* = 4,359	36.2% (24.2, 50.1) *n* = 117	54.0% (45.9, 61.8) *n* = 407	31.0% (22.5, 41.0) *n* = 201
Rao-Scott Adjusted *F*-Test	*F*(1.8, 195.2) = 46.5, *p* < .001			*F*(1.9, 209.2) = 67.9, *p* < .001			
**Past-Year Other Drug Use**^**a**^	2.9% (2.2, .3.8) *n* = 4,697	5.9% (2.7, .12.4) *n* = 144	9.7% (6.2, 14.8) *n* = 459	2.1% (1.5, 2.8) *n* = 4,361	3.8% (1.5, 9.3) *n* = 119	13.5% (8.6, 20.5) *n* = 409	3.4% (1.6, 6.9) *n* = 201
Rao-Scott Adjusted *F*-Test	*F*(1.9, 203.5) = 16.4, *p* < .001			*F*(1.6, 167.9) = 44.1, *p* < .001			
**Married**	46.7% (44.2, 49.2) *n* = 4,696	5.1% (2.0, 12.7) *n* = 139	20.8% (16.1, 26.4) *n* = 459	45.1% (42.6, 47.5) *n* = 4,360	3.5% (0.8, 14.4) *n* = 114	22.5% (16.9, 29.3) *n* = 409	32.0% (21.7, 44.4) *n* = 201
Rao-Scott Adjusted *F*-Test	*F*(2.0, 214.4) = 54.3, *p* < .001			*F*(2.0, 213.5) = 31.9, *p* < .001			
**Number of Times Married (Mean)**	0.7 (0.7, 0.8) *n* = 4,698	0.3 (0.1, 0.4) *n* = 44	0.5 (0.4, 0.6) *n* = 459	0.7 (0.6, 0.7) *n* = 4,362	0.2 (0.1, 0.4) *n* = 120	0.4 (0.3, 0.5) *n* = 409	0.4 (0.3, 0.5) *n* = 201
Design-Adjusted *F*-Test	*F*(2, 107) = 30.4, *p* < .001			*F*(2, 107) = 18.1, *p* < .001			
**Household Size (Mean)**	3.5 (3.5, 3.6) *n* = 4,698	3.0 (2.7, 3.3) *n* = 144	3.3 (3.1, 3.5) *n* = 459	3.5 (3.4, 3.6) *n* = 4,362	2.7 (2.3, 3.1) *n* = 120	3.3 (3.0, 3.5) *n* = 409	3.5 (3.1, 3.8) *n* = 201
Design-Adjusted *F*-Test	*F*(2, 107) = 9.8, *p* < .001			*F*(2, 107) = 7.6, *p* < .001			
**Children Under 18 in HH**	58.7% (56.4, 61.1) *n* = 4,698	29.6% (19.5, 42.2) *n* = 144	37.1% (31.4, 43.2) *n* = 459	56.5% (53.6, 59.2) *n* = 4,362	23.2% (12.21, 39.5) *n* = 120	35.8% (29.2, 42.9) *n* = 409	48.8% (38.7, 58.9) *n* = 201
Rao-Scott Adjusted *F*-Test	*F*(1.9, 208.1) = 30.9, *p* < .001			*F*(1.9, 208.6) = 20.8, *p* < .001			
**Want a/another Child**	49.5% (46.9, 52.1) *n* = 4,563	39.2% (28.3, 51.3) *n* = 138	53.2% (46.4, 59.9) *n* = 446	50.7% (47.7, 53.8) *n* = 4,239	53.5% (40.0, 66.5) *n* = 115	68.1% (61.6, 74.0) *n* = 398	49.3% (38.6, 60.0) *n* = 197
Rao-Scott Adjusted *F*-Test	*F*(2.0, 215.5) = 1.97, *p* = .142			*F*(2.0, 215.8) = 11.7, *p* < .001			
**Currently Pregnant**	3.7% (2.9, 4.7) *n* = 4,011	0% *n* = 123	2.7% (1.4, 5.1) *n* = 433	4.5% (3.5, 5.8) *n* = 3,769	0.3% (0.0, 2.4) *n* = 106	2.5% (1.3, 4.8) *n* = 379	4.1% (1.2, 13.3) *n* = 186
Rao-Scott Adjusted *F*-Test	*F*(1.7, 187.9) = 1.4, *p* = .251			*F*(1.5, 162.9) = 4.7, *p* < .05			
**Lifetime Contraceptive Use**	88.1% (86.6, 89.5) *n* = 4,698	53.8% (41.4, 65.7) *n* = 144	85.9% (80.3, 90.1) *n* = 459	86.3% (84.4, 88.0) *n* = 4,362	53.1% (39.6, 66.2) *n* = 120	85.8% (78.8, 90.7) *n* = 409	69.2% (57.2, 79.0) *n* = 201
Rao-Scott Adjusted *F*-Test	*F*(2.0, 212.6) = 19.2, *p* < .001			*F*(2.0, 211.6) = 19.2, *p* < .001			
**Past-Year Contraceptive Use**	39.5% (37.4, 41.6) *n* = 4,473	23.0% (11.7, 40.2) *n* = 110	43.3% (36.2, 50.6) *n* = 424	38.9% (36.2, 41.5) *n* = 4,134	18.5% (9.5, 32.9) *n* = 86	49.0% (41.4, 56.7) *n* = 386	30.6% (22.7, 39.8) *n* = 176
Rao-Scott Adjusted *F*-Test	*F*(1.9, 204.1) = 2.8, *p* < .10			*F*(1.9, 203.4) = 8.2, *p* < .001			
**Lifetime Sexual Activity, No Contraceptive Use**	9.2% (8.0, 10.7) *n* = 4,492	44.2% (31.6, 57.6) *n* = 140	11.9% (7.9, 17.6) *n* = 441	10.6% (9.2, 12.1) *n* = 4,154	44.5% (31.6, 58.2) *n* = 116	11.5% (6.8, 18.8) *n* = 393	27.6% (17.3, 41.1) *n* = 183
Rao-Scott Adjusted *F*-Test	*F*(2.0, 213.0) = 36.8, *p* < .001			*F*(1.9, 207.0) = 22.6, *p* < .001			
**Past-Year Sexual Activity, No Contraceptive Use**	59.3% (57.2, 61.4) *n* = 3,967	75.1% (57.1, 87.3) *n* = 103	54.4% (47.0, 61.6) *n* = 400	60.1% (57.3, 62.8) *n* = 3,691	80.7% (65.3, 90.3) *n* = 80	50.3% (42.1, 58.6) *n* = 353	70.3% (59.9, 79.0) *n* = 147
Rao-Scott Adjusted *F*-Test	*F*(1.9, 200.6) = 2.8, *p* < .10			*F*(1.9, 203.6) = 7.0, *p* < .01			
**Number of Lifetime Sexual Partners (Mean)**	4.3 (4.2, 4.4) *n* = 4,648	6.7 (5.5, 7.9) *n* = 144	7.6 (7.0, 8.1) *n* = 458	4.3 (4.1, 4.5) *n* = 4,323	7.2 (6.1, 8.4) *n* = 119	7.4 (6.9, 7.9) *n* = 408	4.8 (4.0, 5.7) *n* = 195
Design-Adjusted *F*-Test	*F*(2, 107) = 70.2, *p* < .001			*F*(2, 107) = 81.6, *p* < .001			
**Number of Past-Year Sexual Partners (Mean)**	1.1 (1.0, 1.1) *n* = 4,673	1.3 (1.0 1.6) *n* = 144	1.7 (1.5, 1.9) *n* = 458	1.1 (1.0, 1.1) *n* = 4,335	1.3 (1.1, 1.4) *n* = 119	1.7 (1.6, 1.9) *n* = 408	1.2 (1.0, 1.4) *n* = 196
Design-Adjusted *F*-Test	*F*(2, 107) = 22.8, *p* < .001			*F*(2, 107) = 28.0, *p* < .001			
**Lifetime Report of Anal Sex**	34.8% (32.5, 37.2) *n* = 4,661	23.4% (12.5, 39.4) *n* = 144	58.8% (51.8, 65.5) *n* = 459	34.8% (32.2, 37.5) *n* = 4,333	13.9% (5.8, 29.6) *n* = 119	60.9% (54.5, 67.0) *n* = 408	33.1% (23.4, 44.5) *n* = 200
Rao-Scott Adjusted *F*-Test	*F*(1.8, 193.6) = 17.7, *p* < .001			*F*(1.8, 196.2) = 28.4, *p* < .001			
**Ever had an STD**	14.9% (13.3, 16.6) *n* = 4,692	4.5% (2.3, 8.5) *n* = 144	15.8% (11.9, 20.8) *n* = 458	13.1% (11.7, 14.6) *n* = 4,359	17.0% (6.1, 39.3) *n* = 119	18.4% (13.7, 24.3) *n* = 409	16.2% (8.5, 28.7) *n* = 201
Rao-Scott Adjusted *F*-Test	*F*(1.8, 191.5) = 5.8, *p* = .005			*F*(1.6, 174.0) = 1.5, *p* = .230			
**Past-Year Report of an STD**	1.8% (1.2, 2.6) *n* = 4,692	2.1% (0.6, 6.9) *n* = 144	6.7% (3.6, 12.3) *n* = 458	1.4% (1.1, 1.8) *n* = 4,360	0.3% (0.04, 2.0) *n* = 119	3% (1.9, 4.8) *n* = 409	3.7% (1.4, 9.5) *n* = 201
Rao-Scott Adjusted *F*-Test	*F*(1.7, 184.9) = 10.4, *p* < .001			*F*(1.7, 183.0) = 7.3, *p* < .01			

^a^Other drug use includes the use of cocaine, crack, and methamphetamines

In Table 4, consider the four outcomes measuring past-year pack-a-day smoker, past-year marijuana use, wanting a/another child, and ever had an STD. The estimates suggest that females provided with the “something else” response option for sexual identity (TG2) tend to have *lower* prevalence of these four outcomes, relative to those identifying as lesbian or bisexual when given the four possible options. Table A9 in the online Appendix shows that some of these differences (e.g., past-year marijuana use) remain robust in multivariable models fitted to the TG2 data. Accordingly, we see reductions in the prevalence estimates for lesbian and bisexual respondents based on the sample given the three-category version of sexual identity, where respondents are forced to choose from one of the three identities. In some cases (past-year pack-a-day smoker, wanting a/another child), this leads to non-significant associations in TG1, relative to significant associations in TG2. In others, a non-significant association in TG2 becomes significant in TG1 (ever had an STD). In the case of past-year marijuana use, we see evidence of a larger bisexual-lesbian difference in TG2.

Consider the indicator of wanting a/another child for females. With the four-category measure of sexual identity, there is evidence of a larger difference between bisexual women and heterosexual women, with an estimated 68.1% of bisexual women wanting a/another child (compared to only 50.7% of heterosexual women). This produces a significant Rao-Scott test of association (*p* < .001), suggesting significant differences in the prevalence of this outcome between subgroups defined by sexual identity. The estimated prevalence for a female identifying as “something else” is only 49.3%, and Table A9 in the online Appendix suggests that this subgroup has a marginally lower prevalence than the other three groups when adjusting for other covariates. When we consider the estimates based on the sample assigned to the three-category measure of sexual identity, the estimated prevalence drops for both lesbian and bisexual respondents (especially so for bisexual respondents), to the point where there is no longer evidence of a significant association between sexual identity and “wantedness” (*p* = .142).

When following our multivariable modeling approach to determine which of these changes in associations remained large in magnitude after adjusting for relevant covariates, we identified three outcomes where the models had non-zero two-way interactions between sexual identity and the measurement approach (TG1 vs. TG2). These included past-year marijuana use, wanting a/another child, and ever had an STD. See Table A6 in the online Appendix for the estimated coefficients, 95% CIs, and corresponding design-adjusted Wald tests. Based on the model for past-year marijuana use, the 95% CIs for the odds ratios comparing lesbian and bisexual individuals to heterosexual individuals in TG1 both did not include 1, suggesting large differences based on TG1 (odds ratios of 2.3 (95% CI = 1.5, 3.4) and 2.5 (95% CI = 1.9, 3.5), respectively). Based on TG2, the estimated odds ratio comparing bisexual individuals to heterosexual individuals was 4.5 (95% CI = 3.2, 6.4), suggesting a much larger adjusted difference between heterosexuals and bisexuals when using the four-category measure of sexual identity (consistent with Table 4).

Based on the model for wanting a/another child, the estimated odds ratios for lesbian and bisexual individuals versus heterosexual individuals were both less than 1, with 95% CIs that did not include 1 (OR = 0.3 (95% CI = 0.2, 0.6) and OR = 0.5 (95% CI = 0.3, 0.7), respectively), suggesting reductions in the odds of wanting a/another child for sexual minority females relative to heterosexual females (consistent with the lesbian vs. heterosexual difference in Table 4, but different for bisexuals after adjustment for covariates). In contrast, the 95% CIs both included one for TG2, suggesting negligible differences. We would again arrive at completely different conclusions about these differences after adjusting for the covariates, depending on the measurement approach; the estimates of the adjusted differences shrink and become positive when using the four-category measure of sexual identity.

Based on the multivariable model for ever had an STD, the estimated odds of ever having had an STD are about 70% lower for lesbian women relative to heterosexual women (OR = 0.3, 95% CI = 0.2, 0.6), adjusting for the covariates, while the estimated odds are about 40% higher for bisexual women relative to heterosexual women (OR = 1.4, 95% CI = 1.0, 2.0). In TG2, the estimated lesbian-heterosexual difference disappears (OR = 1.4, 95% CI = 0.4, 4.6), and the bisexual-heterosexual difference remained large, even increasing (OR = 2.0, 95% CI = 1.3, 3.0). We would reach a different conclusion about the adjusted lesbian-heterosexual difference depending on the measurement approach.

**Fig. 2:**
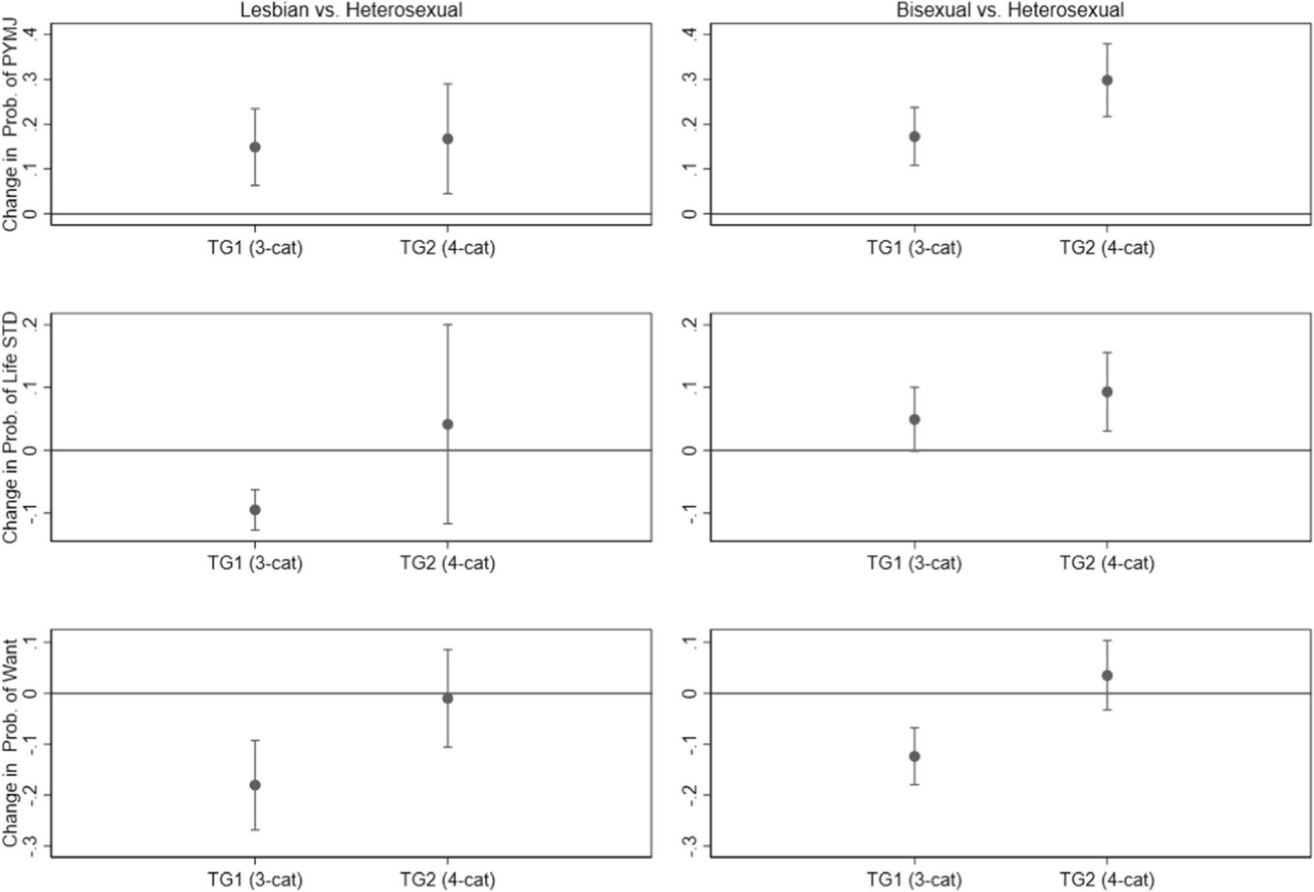
Estimates of marginal differences between lesbian and heterosexual females (first column) and bisexual and heterosexual females (second column) in the probabilities of selected outcomes by measurement approach (TG1 = three categories of sexual identity, TG2 = four categories of sexual identity), including 95% CIs for the marginal differences, based on estimated multivariable models (PYMJ = past-year marijuana use; Life STD = ever had an STD; Want = want a/another child)

Given these modeling results, Fig. 2 displays estimates of the marginal differences between lesbian and heterosexual individuals (first column) and between bisexual and heterosexual individuals (second column) in terms of these three outcomes (adjusting for the covariates in the models), along with 95% CIs for the marginal differences. Figure 2 provides another clear visualization of these estimated differences depending on the measurement approach, where again in one case a 95% CI for the difference may include zero, and in a second case it does not.

In none of our comparative or multivariable analyses did our primary conclusions change when conducting the multiple imputation analysis. None of the significance levels in any of the Rao-Scott tests changed in any meaningful fashion, and we found the same evidence of meaningful interactions in the multivariable models. No new two-way interactions with non-zero coefficients emerged in the multiple imputation analysis.

## Discussion

### Summary of Results

First, among males, we would arrive at different conclusions regarding sexual identity subgroup differences for 3 out of the 19 health outcomes considered (16% of the measures). While many of the measures may not be affected, there could be important policy implications when making conclusions about these populations based on the three measures that were affected (illicit drug use, household size, and cigarette smoking). Comparing bisexual males to heterosexual males, the three-category measure produced a meaningful positive difference for other illicit drug use that disappeared when using four categories, largely because the “something else” respondents had a higher prevalence on this measure, eliminating the large positive difference between heterosexual and bisexual individuals in the three-category version. The four-category measure produced a meaningful negative difference between bisexual and heterosexual males for past-year cigarette use, again because the high prevalence for “something else” respondents appeared to increase the prevalence for bisexual respondents in the three-category version, in this case eliminating the large difference between the three groups that we observed when using the four-category measure. For household size, both gay and bisexual men had lower means when using the three-category version of the measure, and these differences disappeared when using the four-category measure.

Among females, we would arrive at different conclusions regarding sexual identity subgroup differences for 3 out of 20 health outcomes (15% of the measures: wanting a/another child, ever had an STD, and past-year marijuana use). Meaningful negative differences between both lesbian and bisexual women and heterosexual women in the probability of wanting a/another child when using the three-category version of sexual identity disappeared when using the four-category version, in this case because “something else” individuals had a lower prevalence of this outcome that appeared to reduce the prevalence for lesbian and bisexual women when using the three-category measure. In terms of ever having had an STD, a large negative difference between lesbian women and heterosexual women in the three-category version disappeared when using the four-category version, while a negligible difference between bisexual women and heterosexual women because a large positive difference in the four-category version. This all may have been due to lower prevalence of this outcome among women identifying as “something else”. Finally, while large positive differences were found between both lesbian and bisexual women and heterosexual women in terms of past-year marijuana use when using both versions of the sexual identity measure, the difference between bisexual and heterosexual women was found to become substantially larger when using the four-category version (again possibly due to lower prevalence of this outcome among women identifying as “something else”).

These results suggest key sex differences in the studied behaviors among members of the “something else” group (TG2) that was administered the four-category sexual identity measure. In males, these individuals had relatively high rates of not just illicit drug use and cigarette use, but also binge alcohol use. In contrast, the “something else” group of females had lower rates of substance use that were often like those of heterosexual females. At least one longitudinal study has shown that males who reported sexual identity as “something else” had lower odds of cigarette (re)uptake than heterosexual males; this association was not mediated by internalizing and externalizing symptoms (Evans-Polce et al., 2022). Research in Australian sexual minority adults (McLaren, 2015) suggests that internalized homonegativity has stronger links to depressive symptoms in men than women, and men who identify as “something else” to ameliorate minority stress and internalized homonegativity may be a higher risk group. In women, however, lesser identification with the sexual minority group or openness about one’s sexual identity has been linked to better mental health (Kuyper & Fokkema, 2011; McLaren et al., 2013), which may partially explain why this group has lower rates of substance use and warrants additional attention.

### Implications for Research and Practice

This study has broad implications for other secondary analyses of existing public health data, given the other major national studies that have measured sexual identity using a small number of categories (Table 1). Considering the results of this study, we do not find that inferences about the associations between sexual identity and all health outcomes are affected by how sexual identity is measured. However, a non-trivial number of estimated associations are indeed impacted by this problem, meaning that policy and programming decisions based on surveys that only offer a small number of response options for sexual identity should be considered carefully (and ideally, associations should be replicated using an independent data source providing more possible options for this construct).

Our hope is that the new federal guidelines mentioned in the Introduction will yield improved measures of sexual identity in all future national health surveys. We would endorse the best practices summarized in the NASEM report described in the Introduction (NASEM, 2022), which includes open-ended measures for those who would describe themselves as “something else”; careful qualitative analysis of such responses would make attenuated or potentially biased estimates of associations less likely. Indeed, a recent qualitative study of sexual and gender minority individuals suggested that current measures are not as clear as they should be and do not allow for sufficient fluidity and complexity in terms of the construct of sexual orientation identity (Suen et al., 2020).

### Future Research Directions

We did not repeat these analyses for different socio-demographic subgroups of males and females (e.g., race/ethnicity). Future work in this area could investigate whether these changes in associations tend to disproportionately affect socio-demographic subgroups of either sex. The experimental design used in the NSFG also did not allow us to answer the question of how people would respond when asked both types of sexual identity questions. In other words, how do the “something else” respondents for each sex tend to answer when forced to choose between a smaller number of categories? Future experiments could examine this further; for instance, one prior study found that people may consider themselves as “mostly” belonging to a particular identity, and these identities could easily change depending on the response options provided (McCabe et al., 2012).

For example, it is possible that some people who would consider themselves “mostly heterosexual”, representing a unique but understudied sexual identity population that has elevated risk of substance use/mental health outcomes (Hughes et al., 2010, 2015; McCabe et al., 2005, 2012; Talley et al., 2016), would respond as heterosexual when only given three sexual identity options but as “something else” when given four options, affecting estimated differences in rates of substance use. We did see reductions in the estimated percentages of the target population identifying as heterosexual when the “something else” option was added (Table 2), which could have also affected the subgroup differences reported in this study. Tables A3a and A3b in the online Appendix present estimated socio-demographic distributions for each of the three largest sexual identity subpopulations (separately for males and females) based on the two measurement approaches and suggest that changes in Hispanic ethnicity and socio-economic status for gay/lesbian and bisexual individuals may be introduced when using the four-category measure of sexual identity (especially for males). How estimated differences in terms of family formation, rather than substance use, might be affected remains unclear and is worthy of future research. Our results suggest that males choosing the “something else” category tend to have a higher prevalence of substance use and average household sizes, while females choosing the “something else” category have less desire to have a/additional children (see Shenkman & Abramovitch, 2021), lower rates of STDs (see Gurnik et al., 2023 and Everett et al., 2019a, 2019b), and reduced odds of past-year marijuana use.

Other recent work has suggested that same-sex male couples may not provide reliable reports of sexual behavior, and this type of measurement error in sexual activity may affect time-varying reports of sexual identity if activity is considered when reporting identity (Walsh & Stephenson, 2021). Future studies could measure sexual identity in different ways for the same individuals over time and collect additional qualitative data to understand important contextual factors that influence these reports of identity, especially considering recent evidence from national longitudinal data showing significant associations between substance use and sexual identify fluidity/stability (Evans-Polce et al., 2023a, 2023b). This would also enable assessment of comprehension difficulties with the terms “homosexual” and “heterosexual” (Ridolfo et al., 2012), and how individuals identifying as one of these categories respond when different approaches are used to measure sexual identity in the future (e.g., “Straight, that is, not gay or lesbian”).

### Supplementary Information

Below is the link to the electronic supplementary material.

Supplementary file1 (DOCX 68 kb)

## Data Availability

All data analyzed are available in the public domain at the respective NSFG (https://www.cdc.gov/nchs/nsfg/) and NSDUH (https://www.samhsa.gov/data/data-we-collect/nsduh-national-survey-drug-use-and-health) websites.

## Footnotes

1 We note that similar associations were found when conducting analyses of associations between the same three-category measure of sexual identity and common substance use outcomes in the 2015-2019 NSDUH data (for both males and females; SAMHSA, 2018a, 2018b, 2018c, 2019, 2020). This supports the idea that similar conclusions would arise when using the same three-category measure of sexual identity in different national surveys of the same target population, and the NSDUH is considered a gold-standard national survey of substance use. Please see Tables A1 and A2 in the online Appendix for details.

## Code Availability

All of the code used to perform the data management and analyses presented in this manuscript is available in the following GitHub repository: https://github.com/bradytwest/sexident.
