# Supplementary material for: How a “Something Else” Response Option for Sexual Identity Affects National Survey Estimates of Associations Between Sexual Identity, Reproductive Health, and Substance Use
Source: Arch Sex Behav. 2023 Oct 18;53(1):107–26. doi: 10.1007/s10508-023-02710-7 (PMC10794379; doi:10.1007/s10508-023-02710-7)
Supplement: Supplementary file 1 — Supplementary file1 (DOCX 68 kb) [file 10508_2023_2710_MOESM1_ESM.docx]

**Appendix for “How a ‘Something Else’ Response Option for Sexual Identity Affects National Survey Estimates of Associations Between Sexual Identity, Reproductive Health,**

**and Substance Use”**

**Comparing Estimated Differences Based on NSFG-TG1 and the NSDUH**

The following two tables (Tables A1 and A2) present model fitting results testing whether or not the differences in associations between the three-category measures of sexual identity and the 17 common health outcomes (8 for males, 9 for females) were significant when comparing the NSFG to the National Survey of Drug Use and Health (NSDUH). The NSDUH is widely considered to be a gold standard national survey of drug use in the U.S. (for design details, see <https://www.samhsa.gov/data/data-we-collect/nsduh-national-survey-drug-use-and-health>). We wanted to see if a large national survey using a three-category sexual identity measurement approach identical to that of TG1 in the NSFG was generally producing similar estimated differences in terms of common outcome measures between the three sexual identity subgroups. In general, we found hardly any evidence of significant differences in these associations, as none of the interactions between survey and sexual identity emerged as significant at the 0.01 level (which was used given the number of tests performed). We also found similarity in the weighted distributions of various socio-demographic characteristics for the target population between the ages of 18 and 49 based on NSDUH and TG1 of the NSFG; this is not surprising, given the similar weighting approaches that are employed by the two studies.

**Table A1:** Interactions of Survey Instrument (NSFG vs. NSDUH) and Three-Category Measure of Sexual Identity for Outcome Variables of Interest among Males.

|  | Past-Month Binge Drinking | Past-Year Cigarette Use | Past-Year Pack-a-Day Smoker | Past-Year Marijuana Use | Past-Year Other Drug Use^a^ | Past-Year STD Status |
| --- | --- | --- | --- | --- | --- | --- |
|  | Coef. (95% CI) | Coef. (95% CI) | Coef. (95% CI) | Coef. (95% CI) | Coef. (95% CI) | Coef. (95% CI) |
|  | n = 82,688 | n = 82,711 | n = 82,711 | n = 82,653 | n = 82,708 | n = 82,431 |
| **Survey Instrument** |  |  |  |  |  |  |
| NSDUH | REF | REF | REF | REF | REF | REF |
| NSFG | 0.2 (0.1, 0.3)*** | -0.2 (-0.3 -0.0)* | 0.5 (0.3, 0.7)*** | 0.2 (0.1, 0.3)*** | 0.1 (-0.1, 0.3) | -1.3 (-1.8, -0.9)*** |
| **Sexual Identity** |  |  |  |  |  |  |
| Heterosexual | REF | REF | REF | REF | REF | REF |
| Gay | 0.2 (0.0, 0.3)** | 0.2 (0.1, 0.4)*** | 0.1 (-0.2, 0.4) | 0.7 (0.5, 0.8)*** | 1.1 (0.8, 1.3)*** | 1.9 (1.7, 2.1)*** |
| Bisexual | -0.1 (-0.2, 0.0)^†^ | 0.2 (0.1, 0.4)** | 0.1 (-0.1, 0.4) | 0.5 (0.4, 0.7)*** | 0.5 (0.3, 0.7)*** | 1.0 (0.7, 1.3)*** |
| **Survey Instrument x Sexual Identity** |  |  |  |  |  |  |
| NSDUH x Gay | -0.1 (-0.6, 0.4) | -0.3 (-0.9, 0.2) | 0.1 (-0.7, 0.9) | 0.3 (-0.2, 0.8) | -0.2 (-1.0, 0.6) | 0.8 (-0.1, 1.7)^†^ |
| NSDUH x Bisexual | -0.0 (-0.7, 0.6) | 0.1 (-0.5, 0.7) | 0.1 (-0.7, 1.0) | -0.0 (-0.6, 0.5) | 0.7 (0.0, 1.5)* | 0.9 (-0.3, 2.2) |
| **Adj. Wald F-Test** | F(2, 157) = 0.14 | F(2, 157) = 0.66 | F(2, 157) = 0.07 | F(2, 157) = 0.83 | F(2, 157) = 2.34^†^ | F(2, 157) = 2.24 |
| **Area Under the Curve** | AUC = 0.597 | AUC = 0.685 | AUC = 0.790 | AUC = 0.661 | AUC = 0.681 | AUC = 0.632 |

^†^P< 0.10, *P < 0.05, **P < 0.01, ***P < 0.001

All models control for race, age, educational attainment, and household income.

^a^Other drug use includes the use of cocaine, crack, and methamphetamines.

**Table A1**, Cont.

|  | Household Size | Child/Children under 18 in Household |
| --- | --- | --- |
|  | Coef. (95% CI) | Coef. (95% CI) |
|  | n = 82,713 | n = 82,713 |
| **Survey Instrument** |  |  |
| NSDUH | REF | REF |
| NSFG | -0.1 (-0.1, 0.0)^†^ | -0.3 (-0.4, -0.2)*** |
| **Sexual Identity** |  |  |
| Heterosexual | REF | REF |
| Gay | -0.8 (-0.8, -0.7)*** | -1.7 (-1.9, -1.6)*** |
| Bisexual | -0.1 (-0.3, -0.0)* | -0.5 (-0.6, -0.3)*** |
| **Survey Instrument x Sexual Identity** |  |  |
| NSDUH x Gay | 0.1 (-0.3, -0.4) | -0.6 (-2.0, 0.7) |
| NSDUH x Bisexual | -0.4 (-0.7, -0.1)* | -1.0 (-1.6, -0.3)** |
| **Adj. Wald F-Test** | F(2, 157) = 3.23* | F(2, 157) = 4.49* |
| **Area Under the Curve / R-squared** | R^2^ = 0.096 | AUC = 0.667 |

^†^P< 0.10, *P < 0.05, **P < 0.01, ***P < 0.001

All models control for race, age, educational attainment, and household income.

**Table A2:** Interactions of Survey Instrument (NSFG vs. NSDUH) and Three-Category Measure of Sexual Identity for Outcome Variables of Interest among Females.

|  | Past-Month Binge Drinking | Past-Year Cigarette Use | Past-Year Pack-a-Day Smoker | Past-Year Marijuana Use | Past-Year Other Drug Use^a^ | Past-Year STD Status |
| --- | --- | --- | --- | --- | --- | --- |
|  | Coef. (95% CI) | Coef. (95% CI) | Coef. (95% CI) | Coef. (95% CI) | Coef. (95% CI) | Coef. (95% CI) |
|  | n = 94,032 | n = 94,051 | n = 94,051 | n = 94,008 | n = 94,048 | n = 93,792 |
| **Survey Instrument** |  |  |  |  |  |  |
| NSDUH | REF | REF | REF | REF | REF | REF |
| NSFG | 0.2 (0.1, 0.3)** | -0.1 (-0.2, 0.1) | 0.3 (0.0, 0.5)* | 0.2 (0.1, 0.3)** | 0.2 (-0.1, 0.5) | -0.5 (-0.9, -0.1)** |
| **Sexual Identity** |  |  |  |  |  |  |
| Heterosexual | REF | REF | REF | REF | REF | REF |
| Lesbian/Gay | 0.4 (0.3, 0.6)*** | 0.8 (0.6, 0.9)*** | 0.4 (0.1, 0.7)** | 0.9 (0.8, 1.0)*** | 0.6 (0.3, 0.9)*** | -0.6 (-1.0, -0.2)** |
| Bisexual | 0.4 (0.3, 0.5)*** | 0.8 (0.7, 0.9)*** | 0.5 (0.3, 0.7)*** | 1.1 (1.0, 1.2)*** | 1.1 (0.9, 1.2)*** | 0.6 (0.5, 0.8)*** |
| **Survey Instrument x Sexual Identity** |  |  |  |  |  |  |
| NSDUH x Lesbian/Gay | 0.1 (-0.5, 0.7) | -0.3 (-0.8, 0.2) | -0.2 (-1.0, 0.6) | -0.1 (-0.5, 0.4) | -0.1 (-1.0, 0.8) | 0.6 (-0.8, 2.0) |
| NSDUH x Bisexual | -0.0 (-0.3, 0.3) | -0.2 (-0.5, 0.2) | 0.0 (-0.6, 0.7) | -0.2 (-0.5, 0.2) | -0.1 (-0.6, 0.5) | 0.6 (-0.2, 1.3) |
| **Adj. Wald F-Test** | F(2, 157) = 0.07 | F(2, 157) = 0.80 | F(2, 157) = 0.11 | F(2, 157) = 0.67 | F(2, 157) = 0.07 | F(2, 157) = 1.33 |
| **Area Under the Curve** | AUC = 0.590 | AUC = 0.686 | AUC = 0.899 | AUC = 0.662 | AUC = 0.682 | AUC = 0.597 |

^†^P< 0.10, *P < 0.05, **P < 0.01, ***P < 0.001

All models control for race, age, educational attainment, and household income.

^a^Other drug use includes the use of cocaine, crack, and methamphetamines.

**Table A2**, Cont.

|  | Household Size | Child/Children under 18 in Household | Currently Pregnant |
| --- | --- | --- | --- |
|  | Coef. (95% CI) | Coef. (95% CI) | Coef. (95% CI) |
|  | n = 94,051 | n = 94,051 | n = 82,993 |
| **Survey Instrument** |  |  |  |
| NSDUH | REF | REF | REF |
| NSFG | -0.1 (-0.2, -0.0)** | -0.2 (-0.3, -0.1)*** | -0.1 (-0.3, 0.2) |
| **Sexual Identity** |  |  |  |
| Heterosexual | REF | REF | REF |
| Gay | -0.5 (-0.6, -0.4)*** | -1.0 (-1.1, -0.8)*** | -2.2 (-3.1, -1.4)*** |
| Bisexual | -0.1 (-0.1, -0.0)** | -0.2 (-0.3, -0.2)*** | -0.4 (-0.6, -0.2)*** |
| **Survey Instrument x Sexual Identity** |  |  |  |
| NSDUH x Lesbian/Gay | 0.1 (-0.2, 0.4) | -0.2 (-0.7, 0.4) | EMPTY |
| NSDUH x Bisexual | -0.1 (-0.3, 0.1) | -0.4 (-0.6, -0.1)** | -0.1 (-0.9, 0.7) |
| **Adj. Wald F-Test** | F(2, 157) = 1.36 | F(2, 157) = 4.17* | F(1, 158) = 0.06 |
| **Area Under the Curve / R-squared** | R^2^ = 0.078 | AUC = 0.658 | AUC = 0.654 |

^†^P< 0.10, *P < 0.05, **P < 0.01, ***P < 0.001

All models control for race, age, educational attainment, and household income.

**Estimated Socio-Demographic Distributions of Each Sexual Identity Subgroup**

Tables A3a and A3b present weighted estimates of the distributions of selected socio-demographic features (including means for age) for the three largest sexual identity subpopulations in the target NSFG population, based on the half-samples randomly assigned to each sexual identity measurement approach. Considering males first (Table A3a), while the estimated distributions for heterosexuals are quite similar, we see that when using the four-category version of sexual identity, the gay population is estimated to be slightly less Hispanic, less White, and having higher income, while the bisexual population is estimated to be slightly more White, more Hispanic, less-educated, and younger. These slight differences suggest that the measurement approach may also shift the socio-demographic features of individuals selecting each response option for sexual identity.

**Table A3a:** Estimated socio-demographic distributions for sexual identity subgroups of males based on the two sexual identity measurement approaches evaluated in the NSFG (2015-2019).

| Males | Treatment Group 1 (Three Categories) | | | Treatment Group 2 (Four Categories) | | |
| --- | --- | --- | --- | --- | --- | --- |
| Socio-demographic feature | Heterosexual | Gay | Bisexual | Heterosexual | Gay | Bisexual |
| Race |  |  |  |  |  |  |
| White | 76.8% | 77.6% | 77.8% | 75.7% | 70.6% | 83.2% |
| Black | 14.3% | 8.1% | 11.8% | 15.5% | 8.2% | 11.6% |
| Other | 8.8% | 14.3% | 10.4% | 8.8% | 21.2% | 5.2% |
| Hispanic |  |  |  |  |  |  |
| No | 79.2% | 63.9% | 81.0% | 79.6% | 73.3% | 75.6% |
| Yes | 20.8% | 36.2% | 19.0% | 20.4% | 26.7% | 24.4% |
| Age (Mean) | 33.4 | 31.1 | 28.3 | 33.1 | 34.7 | 20.4 |
| Education |  |  |  |  |  |  |
| Less than high school | 11.1% | 6.9% | 8.6% | 9.0% | 2.8% | 16.1% |
| High school degree or equivalent | 29.4% | 18.0% | 37.3% | 30.4% | 23.1% | 25.6% |
| Greater than high school | 59.5% | 75.1% | 54.1% | 60.7% | 74.1% | 58.4% |
| HH Income |  |  |  |  |  |  |
| $0-19,999 | 14.1% | 15.0% | 20.7% | 11.5% | 17.6% | 19.7% |
| $20,000- 39,999 | 20.5% | 24.7% | 23.7% | 20.5% | 19.9% | 29.6% |
| $40,000-74,999 | 26.0% | 32.3% | 22.5% | 27.7% | 25.9% | 19.0% |
| $75,000+ | 39.4% | 28.0% | 33.1% | 40.3% | 36.6% | 31.7% |

These differences could explain why we found that some of the estimated differences in associations between sexual identity and the outcomes for males were no longer as large when moving from a bivariate analysis to a multivariable analysis (e.g., wanting a/another child in the future). For example, if fewer Whites identify as gay males based on the four-category measurement approach (possibly because they tend to identify as gay if “something else” is not available), and being White increases the probability of wanting a/another child in the future, this could explain why the large change in the heterosexual – gay difference in this probability depending on the measurement approach (Table 3 in the paper) disappears in the multivariable analysis adjusting for race and ethnicity.

Considering women next (Table A3b), we see fewer shifts in the estimated socio-demographic distributions based on the measurement approach. There is some evidence of individuals identifying as gay in the four-category approach tending to have lower education. But unlike males, there was no evidence of large changes in the associations of sexual identity with the outcomes (depending on the measurement approach) disappearing in the multivariable analyses; the same differences in associations observed in the initial bivariate analysis remained robust in the multivariable models.

**Table A3b:** Estimated socio-demographic distributions for sexual identity subgroups of females based on the two sexual identity measurement approaches evaluated in the NSFG (2015-2019).

| Females | Treatment Group 1 (Three Categories) | | | Treatment Group 2 (Four Categories) | | |
| --- | --- | --- | --- | --- | --- | --- |
| Socio-demographic feature | Heterosexual | Gay | Bisexual | Heterosexual | Gay | Bisexual |
| Race |  |  |  |  |  |  |
| White | 75.5% | 74.5% | 74.9% | 75.4% | 78.5% | 77.6% |
| Black | 15.2% | 14.9% | 15.5% | 16.2% | 18.9% | 16.5% |
| Other | 9.3% | 10.7% | 9.6% | 8.4% | 2.5% | 6.0% |
| Hispanic |  |  |  |  |  |  |
| No | 78.9% | 84.6% | 82.2% | 81.1% | 86.3% | 82.4% |
| Yes | 21.2% | 15.4% | 17.8% | 18.9% | 13.7% | 17.6% |
| Age (Mean) | 34.1 | 31.7 | 28.4 | 33.6 | 31.8 | 28.8 |
| Education |  |  |  |  |  |  |
| Less than high school | 9.5% | 9.2% | 11.3% | 7.6% | 13.6% | 9.7% |
| High school degree or equivalent | 23.9% | 25.1% | 25.6% | 25.1% | 28.9% | 29.0% |
| Greater than high school | 66.6% | 65.7% | 63.2% | 67.3% | 57.6% | 61.3% |
| HH Income |  |  |  |  |  |  |
| $0-19,999 | 19.1% | 25.4% | 27.7% | 18.9% | 29.2% | 29.7% |
| $20,000- 39,999 | 21.7% | 28.6% | 27.2% | 21.7% | 18.5% | 25.4% |
| $40,000-74,999 | 26.6% | 23.7% | 25.6% | 26.5% | 23.2% | 25.1% |
| $75,000+ | 32.7% | 22.3% | 19.6% | 33.0% | 29.1% | 19.8% |

**Estimated Coefficients in Multivariable Models**

Table A4 presents the estimated regression coefficients in the multivariable models for selected outcomes for males, along with 95% confidence intervals for the coefficients. These results provide evidence of non-zero interactions between the survey instrument and sexual identity in the multivariable models for these outcomes (for males). For example, the non-zero positive differences in the log-odds of other drug use between both gay (estimated coefficient = 0.8, 95% CI = 0.0, 1.6) and bisexual (estimated coefficient = 1.3, 95% CI = 0.6, 1.9) males and heterosexual males based on the sample assigned to TG1 (consistent with the results observed in Table 3) approach zero when using the four-category measure (estimated coefficient for TG2 x Bisexual interaction = -1.4, 95% CI = -2.5, -0.3, meaning that the net bisexual coefficient for TG2 is estimated to be 1.3 – 1.4 = -0.1). Each model has an acceptable fit based on the AUC or R-squared values.

**Table A4:** Estimated multivariable logistic regression model results for selected male health outcomes with significant two-way interactions between survey instrument and sexual identity.

|  | Past-Year Cigarette Use | Past-Year Other Drug Use^a^ | Household Size |
| --- | --- | --- | --- |
|  | n = 8,402 | n = 8,398 | n = 8,407 |
|  | **Coef. (95% CI)** | **Coef. (95% CI)** | **Coef. (95% CI)** |
| **Survey Instrument** |  |  |  |
| TG1 | REF | REF | REF |
| TG2 | 0.0 (-0.2, 0.2) | 0.1 (-0.1, 0.4) | -0.1 (-0.2, -0.0)** |
| **Sexual Identity** |  |  |  |
| Heterosexual | REF | REF | REF |
| Gay | -0.0 (-0.6, 0.5) | 0.8 (0.0, 1.6)* | -0.7 (-1.1, -0.4)*** |
| Bisexual | 0.3 (-0.3, 0.9) | 1.3 (0.6, 1.9)*** | -0.6 (-0.8, -0.3)*** |
| **Survey Instrument × Sexual Identity** |  |  |  |
| TG2 **×** Gay | 0.4 (-0.4, 1.2) | -0.6 (-1.6, 0.4) | 0.3 (-0.3, 0.9) |
| TG2 **×** Bisexual | -1.2 (-2.1, -0.3)* | -1.4 (-2.5, -0.3)* | 0.6 (0.1, 1.1)* |
| **Adj. Wald F-Test for Interaction** | F(2, 107) = 4.18* | F(2, 107) = 4.46* | F(2, 107) = 3.16* |
| **Area Under the Curve / R-Squared** | AUC = 0.716 | AUC = 0.668 | R^2^ = 0.096 |

^†^p < 0.10, *p < 0.05, **p < 0.01, ***p < 0.001

All models control for race, age, educational attainment, household income, and Hispanic ethnicity.

^a^Other drug use includes the use of cocaine, crack, and methamphetamines.

Table A5 presents the estimated multivariable models for all other outcomes for males, providing evidence of negligible two-way interactions between sexual identity measurement type and sexual identity.

**Table A5:** Estimated multivariable logistic regression model results for selected male health outcomes with non-significant two-way interactions between survey instrument and sexual identity.

|  | Past-Month Binge Drinking | Past-Year Pack-a-Day Smoker | Past-Year Marijuana Use | Lifetime STD Status | Past-Year STD Status |
| --- | --- | --- | --- | --- | --- |
|  | Coef. (95% CI) | Coef. (95% CI) | Coef. (95% CI) | Coef. (95% CI) | Coef. (95% CI) |
|  | n = 8,364 | n = 8,402 | n = 8,369 | n = 8,389 | n = 8,387 |
| **Survey Instrument** |  |  |  |  |  |
| TG1 | REF | REF | REF | REF | REF |
| TG2 | 0.0 (-0.1, 0.1) | -0.2 (-0.4, 0.1) | -0.1 (-0.2, 0.1) | -0.4 (-0.8, -0.0)* | 0.9 (0.2, 1.5)** |
| **Sexual Identity** |  |  |  |  |  |
| Heterosexual | REF | REF | REF | REF | REF |
| Gay | 0.1 (-0.4, 0.5) | 0.3 (-0.4, 1.0) | 1.0 (0.5, 1.5)*** | 2.1 (1.4, 2.8)*** | 3.0 (2.0, 3.9)*** |
| Bisexual | -0.1 (-0.7, 0.4) | 0.3 (-0.5, 1.1) | 0.5 (-0.0, 1.0)^†^ | 1.9 (1.2, 2.6)*** | 2.0 (0.7, 3.2)** |
| **Survey Instrument x Sexual Identity** |  |  |  |  |  |
| TG2 x Gay | 0.1 (-0.7, 0.9) | -0.8 (-2.0, 0.5) | -0.0 (-0.7, 0.7) | -0.0 (-1.0, 1.0) | -1.1 (-2.8, 0.6) |
| TG2 x Bisexual | -0.3 (-1.1, 0.5) | -1.0 (-2.3, 0.3) | -0.1 (-0.9, 0.7) | -0.9 (-2.2, 0.3) | -1.0 (-2.7, 0.7) |
| **Adj. Wald F-Test** | F(2, 107) = 0.31 | F(2, 107) = 2.08 | F(2, 107) = 0.04 | F(2, 107) = 1.07 | F(2, 107) = 1.43 |
| **Area Under the Curve** | AUC = 0.733 | AUC = 0.790 | AUC = 0.636 | AUC = 0.680 | AUC = 0.753 |

^†^P< 0.10, *P < 0.05, **P < 0.01, ***P < 0.001

All models control for race, age, educational attainment, household income, and Hispanic ethnicity.

**Table A5**, Cont.

|  | Ever Had Sex, but No Condom Last Time and No Vasectomy | Ever Vasectomy | Sexual Activity without Vasectomy | Number of Sex Partners Life | Number of Sex Partners in the Last Year | Lifetime Report of Anal Sex |
| --- | --- | --- | --- | --- | --- | --- |
|  | Coef. (95% CI) | Coef. (95% CI) | Coef. (95% CI) | Coef. (95% CI) | Coef. (95% CI) | Coef. (95% CI) |
|  | n = 8,404 | n = 7,384 | n = 7,102 | n = 8,225 | n = 8,327 | n = 8,401 |
| **Survey Instrument** |  |  |  |  |  |  |
| TG1 | REF | REF | REF | REF | REF | REF |
| TG2 | -0.1 (-0.2, 0.0) | 0.1 (-0.2, 0.4) | -0.1 (-0.4, -0.2) | -0.1 (-0.3, 0.1) | -0.0 (-0.1, 0.0) | -0.1 (-0.2, 0.1) |
| **Sexual Identity** |  |  |  |  |  |  |
| Heterosexual | REF | REF | REF | REF | REF | REF |
| Lesbian/Gay | -0.3 (-0.9, 0.2) | EMPTY | EMPTY | 2.0 (1.4, 2.7)*** | 0.6 (0.3, 0.8)*** | 2.8 (2.0, 3.6)*** |
| Bisexual | -1.6 (-2.3, -0.9)*** | -0.3 (-1.5, 0.9) | 0.3 (-0.9, 1.5) | 3.0 (1.7, 4.2)*** | 0.5 (0.1, 0.8)** | 1.6 (0.9, 2.2)*** |
| **Survey Instrument x Sexual Identity** |  |  |  |  |  |  |
| TG2 x Gay | -0.3 (-1.0, 0.4) | EMPTY | EMPTY | -0.1 (-1.0, 0.9) | 0.0 (-0.3, 0.4) | -0.0 (-1.0, 0.9) |
| TG2 x Bisexual | 0.7 (-0.1, 1.5) ^†^ | -0.4 (-2.3, 1.5) | 0.4 (-1.5, 2.3) | -1.2 (-3.0, 0.6) | -0.2 (-0.8, 0.3) | -0.4 (-1.3, 0.6) |
| **Adj. Wald F-Test** | F(2, 107) = 1.92 | F(1, 108) = 0.18 | F(1, 108) = 0.17 | F(2, 107) = 0.90 | F(2, 107) = 0.40 | F(2, 107) = 0.28 |
| **Area Under the Curve / R-squared** | AUC = 0.657 | AUC = 0.815 | AUC = 0.551 | R^2^ = 0.177 | R^2^ = 0.046 | AUC = 0.666 |

^†^P< 0.10, *P < 0.05, **P < 0.01, ***P < 0.001

All models control for race, age, educational attainment, household income, and Hispanic ethnicity.

**Table A5**, Cont.

|  | Children under 18 in Household | R wants a/another Child | Married | Number of Times Married |
| --- | --- | --- | --- | --- |
|  | Coef. (95% CI) | Coef. (95% CI) | Coef. (95% CI) | Coef. (95% CI) |
|  | n = 8,407 | n = 8,183 | n = 8,290 | n = 8,407 |
| **Survey Instrument** |  |  |  |  |
| TG1 | REF | REF | REF | REF |
| TG2 | -0.2 (-0.3, -0.0)* | 0.1 (-0.0, 0.3) | -0.1 (-0.2, 0.1) | -0.0 (-0.0, 0.0) |
| **Sexual Identity** |  |  |  |  |
| Heterosexual | REF | REF | REF | REF |
| Gay | -2.5 (-3.9, -1.1)** | -0.8 (-1.4, -0.2)** | -2.5 (-3.7, -1.2)*** | -0.5 (-0.6, -0.4)*** |
| Bisexual | -1.3 (-2.0, -0.6)*** | -0.6 (-1.3, 0.2) | -0.8 (-1.6, 0.0)^†^ | -0.1 (-0.3, -0.0)* |
| **Survey Instrument x Sexual Identity** |  |  |  |  |
| TG2 x Gay | 0.3 (-1.5, 2.1) | -0.2 (-1.0, 0.5) | OMIT | 0.0 (-0.2, 0.2) |
| TG2 x Bisexual | 0.4 (-0.4, 1.3) | 0.2 (-0.9, 1.4) | 0.0 (-1.0, 1.0) | 0.1 (-0.1, 0.2) |
| **Adj. Wald F-Test** | F(2, 107) = 0.57 | F(2, 107) = 0.27 | F(1, 108) = 0.00 | F(2, 107) = 0.37 |
| **Area Under the Curve / R-squared** | AUC = 0.776 | AUC = 0.810 | AUC = 0.838 | R^2^ = 0.336 |

^†^P< 0.10, *P < 0.05, **P < 0.01, ***P < 0.001

All models control for race, age, educational attainment, household income, and Hispanic ethnicity.

Table A6 presents evidence of non-zero two-way interaction effects in selected models for females based on design-adjusted Wald tests. These results suggest that for these specific outcomes, the differences between the sexual identity subgroups vary significantly depending on the type of sexual identity measure used (adjusting for the covariates). For example, in the model for wanting a/another child, large negative differences in the log-odds of this outcome for lesbian (estimated coefficient = -1.1, 95% CI = -1.6, -0.5) and bisexual (estimated coefficient = -0.7, 95% CI = -1.1, -0.4) females when compared to heterosexual females based on the TG1 sample are each shifted toward zero in a non-zero fashion based on TG2 (estimated coefficient for TG2 x Lesbian interaction = 1.0, 95% CI = 0.2, 1.8; estimated coefficient for TG2 x Bisexual interaction = 0.9, 95% CI = 0.5, 1.4), resulting in net differences that are close to zero based on the TG2 approach. Each model has acceptable fit based on the AUC values.

**Table A6:** Estimated multivariable logistic regression model results for selected female health outcomes with significant two-way interactions between survey instrument and sexual identity.

|  | **Outcome Measure** | | |
| --- | --- | --- | --- |
|  | Past-Year Marijuana Use | Lifetime STD Status | R Wants a/another Child |
|  | n = 10**,**178 | n = 10,181 | n = 9,899 |
|  | **Coef. (95% CI)** | **Coef. (95% CI)** | **Coef. (95% CI)** |
| **Survey Instrument** |  |  |  |
| TG1 | REF | REF | REF |
| TG2 | -0.1 (-0.3, 0.0) | -0.1 (-0.3, 0.0) | -0.0 (-0.2, 0.1) |
| **Sexual Identity** |  |  |  |
| Heterosexual | REF | REF | REF |
| Lesbian | 0.8 (0.4, 1.2)*** | -1.2 (-1.8, -0.5)** | -1.1 (-1.6, -0.5)*** |
| Bisexual | 0.9 (0.6, 1.2)*** | 0.4 (0.0, 0.7)* | -0.7 (-1.1, -0.4)*** |
| **Survey Instrument × Sexual Identity** |  |  |  |
| TG2 × Lesbian | 0.1 (-0.5, 0.8) | 1.5 (0.4, 2.7)** | 1.0 (0.2, 1.8)* |
| TG2 × Bisexual | 0.6 (0.1, 1.0)* | 0.3 (-0.2, 0.8) | 0.9 (0.5, 1.4)*** |
| **Adj. Wald F-Test for Interaction** | F(2, 107) = 3.02^†^ | F(2, 107)= 4.19* | F(2, 107)= 9.80*** |
| **Area Under the Curve** | AUC = 0.637 | AUC = 0.689 | AUC = 0.808 |

^†^p < 0.10, *p < 0.05, **p < 0.01, ***p < 0.001

All models control for race, age, educational attainment, household income, and Hispanic ethnicity.

Table A7 presents the estimated multivariable models for all other outcomes for females, providing evidence of negligible two-way interactions between sexual identity measurement type and sexual identity.

**Table A7:** Estimated multivariable logistic regression model results for selected female health outcomes with non-significant two-way interactions between survey instrument and sexual identity.

|  | Past-Month Binge Drinking | Past-Year Cigarette Use | Past-Year Pack-a-Day Smoker | Past-Year Other Drug Use^a^ | Past-Year STD Status |
| --- | --- | --- | --- | --- | --- |
|  | Coef. (95% CI) | Coef. (95% CI) | Coef. (95% CI) | Coef. (95% CI) | Coef. (95% CI) |
|  | n = 10,164 | n = 10,192 | n = 10,192 | n = 10,189 | n = 10,182 |
| **Survey Instrument** |  |  |  |  |  |
| TG1 | REF | REF | REF | REF | REF |
| TG2 | -0.1 (-0.2, 0.1) | -0.2 (-0.4, -0.1)** | -0.0 (-0.4, 0.3) | -0.4 (-0.8, 0.0) ^†^ | -0.3 (-0.8, 0.2) |
| **Sexual Identity** |  |  |  |  |  |
| Heterosexual | REF | REF | REF | REF | REF |
| Lesbian/Gay | 0.6 (0.0, 1.2)* | 0.5 (-0.0, 1.0)^†^ | 0.2 (-0.6, 1.0) | 0.5 (-0.4, 1.4) | -0.1 (-1.4, 1.3) |
| Bisexual | 0.4 (0.2, 0.7)** | 0.7 (0.3, 1.0)*** | 0.5 (-0.1, 1.2) | 1.0 (0.5, 1.5)*** | 1.0 (0.3, 1.7)** |
| **Survey Instrument x Sexual Identity** |  |  |  |  |  |
| TG2 x Lesbian/Gay | -0.4 (-1.2, 0.4) | 0.2 (-0.6, 1.0) | 1.0 (-0.4, 2.3) | 0.0 (-1.5, 1.5) | -1.7 (-4.2, 0.8) |
| TG2 x Bisexual | 0.2 (-0.2, 0.6) | 0.5 (0.0, 1.0)* | 0.5 (-0.4, 1.4) | 0.7 (-0.2, 1.6) | -0.6 (-1.4, 0.3) |
| **Adj. Wald F-Test** | F(2, 107) = 0.93 | F(2, 107) = 2.21 | F(2, 107) = 1.24 | F(2, 107) = 1.31 | F(2, 107) = 1.36 |
| **Area Under the Curve** | AUC = 0.602 | AUC = 0.720 | AUC = 0.787 | AUC = 0.273 | AUC = 0.744 |

^†^P< 0.10, *P < 0.05, **P < 0.01, ***P < 0.001

All models control for race, age, educational attainment, household income, and Hispanic ethnicity.

^a^Other drug use includes the use of cocaine, crack, and methamphetamines.

**Table A7**, Cont.

|  | Past-Year Contraceptive Use | Sexual Activity without Contraceptive Use in Past Year | Ever Use Contraceptive in Life | Sexual Activity without Contraceptive Use in Life | Number of Sex Partners Life | Number of Sex Partners in the Last Year | Lifetime Report of Anal Sex |
| --- | --- | --- | --- | --- | --- | --- | --- |
|  | Coef. (95% CI) | Coef. (95% CI) | Coef. (95% CI) | Coef. (95% CI) | Coef. (95% CI) | Coef. (95% CI) | Coef. (95% CI) |
|  | n = 9,613 | n = 8,588 | n = 10,192 | n = 9,736 | n = 10,100 | n = 10,137 | n = 10,124 |
| **Survey Instrument** |  |  |  |  |  |  |  |
| TG1 | REF | REF | REF | REF | REF | REF | REF |
| TG2 | -0.1 (-0.2, 0.1) | 0.1 (-0.1, 0.2) | -0.2 (-0.4, 0.0) | 0.2 (-0.1, 0.4) | -0.0 (-0.2, 0.1) | -0.0 (-0.1, 0.0) | -0.0 (-0.1, 0.1) |
| **Sexual Identity** |  |  |  |  |  |  |  |
| Heterosexual | REF | REF | REF | REF | REF | REF | REF |
| Lesbian/Gay | -1.0 (-1.7, -0.3)** | 1.0 (0.3, 1.7)* | -1.9 (-2.5, -1.1)*** | 2.1 (1.4, 2.8)*** | 2.5 (1.3, 3.6)*** | 0.1 (-0.1, 0.4) | -0.5 (-1.3, 0.3) |
| Bisexual | -0.3 (-0.6, -0.0)* | 0.3 (-0.1, 0.6) | 0.1 (-0.3, 0.6) | 0.1 (-0.4, 0.6) | 3.5 (3.0, 4.0)*** | 0.6 (0.4, 0.8)*** | 1.2 (0.9, 1.5)*** |
| **Survey Instrument x Sexual Identity** |  |  |  |  |  |  |  |
| TG2 x Lesbian/Gay | -0.0 (-1.0, 1.0) | 0.1 (-1.0, 1.1) | -0.0 (1.0, 0.9) | -0.0 (-0.9, 0.9) | 0.4 (-1.3, 2.1) | 0.0 (-0.3, 0.4) | -0.7 (-2.0, 0.6) |
| TG2 x Bisexual | 0.3 (-0.1, 0.8) | -0.3 (-0.7, 0.2) | 0.1 (-0.5, 0.8) | -0.2 (-0.9, 0.6) | -0.1 (-0.9, 0.6) | 0.0 (-0.2, 0.3) | 0.1 (-0.3, 0.4) |
| **Adj. Wald F-Test** | F(2, 107) = 1.21 | F(2, 107) = 0.61 | F(2, 107) = 0.07 | F(2, 107) = 0.10 | F(2, 107) = 0.17 | F(2, 107) = 0.08 | F(2, 107) = 0.69 |
| **Area Under the Curve / R-squared** | AUC = 0.725 | AUC = 0.715 | AUC = 0.787 | AUC = 0.575 | R^2^ = 0.193 | R^2^ = 0.076 | AUC = 0.505 |

^†^P< 0.10, *P < 0.05, **P < 0.01, ***P < 0.001

All models control for race, age, educational attainment, household income, and Hispanic ethnicity,

**Table A7**, Cont.

|  | Household Size | Children under 18 in Household | Married | Number of Times Married | Currently Pregnant |
| --- | --- | --- | --- | --- | --- |
|  | Coef. (95% CI) | Coef. (95% CI) | Coef. (95% CI) | Coef. (95% CI) | Coef. (95% CI) |
|  | n = 10,192 | n = 10,192 | n = 10,177 | n = 10.192 | n = 8,698 |
| **Survey Instrument** |  |  |  |  |  |
| TG1 | REF | REF | REF | REF | REF |
| TG2 | -0.0 (-0.1, 0.1) | -0.0 (-0.1, 0.1) | -0.0 (-0.2, 0.2) | -0.0 (-0.1, 0.0) | 0.2 (-0.2, 0.6) |
| **Sexual Identity** |  |  |  |  |  |
| Heterosexual | REF | REF | REF | REF | REF |
| Gay | -0.4 (-0.8, -0.1)** | -1.1 (-1.7, -0.5)*** | -2.9 (-4.0, -1.8)*** | -0.3 (-0.5, -0.2)*** | -2.9 (-4.9, -0.8)** |
| Bisexual | -0.2 (-0.4, -0.0)* | -0.5 (-0.8, -0.2)** | -0.8 (-1.3, -0.4)*** | -0.0 (-0.1, 0.0) | -0.5 (-1.3, 0.2) |
| **Survey Instrument x Sexual Identity** |  |  |  |  |  |
| TG2 x Lesbian/Gay | -0.3 (-0.8, 0.2) | -0.5 (-1.6, 0.6) | -0.6 (-2.5, 1.4) | -0.0 (-0.3, 0.2) | EMPTY |
| TG2 x Bisexual | 0.0 (-0.3, 0.3) | -0.1 (-0.5, 0.3) | 0.2 (-0.5, 0.8) | -0.0 (-0.1, 0.1) | -0.3 (-1.4, 0.8) |
| **Adj. Wald F-Test** | F(2, 107) = 0.84 | F(2, 107) = 0.47 | F(2, 107) = 0.31 | F(2, 107) = 0.11 | F(1, 108) = 0.22 |
| **Area Under the Curve / R-squared** | R^2^ = 0.0861 | AUC = 0.769 | AUC = 0.841 | R^2^ = 0.299 | AUC = 0.640 |

^†^P< 0.10, *P < 0.05, **P < 0.01, ***P < 0.001

All models control for race, age, educational attainment, household income, and Hispanic ethnicity

**Estimated Coefficients in Multivariable Models for TG2 Specifically**

Table A8 presents estimated coefficients in the multivariable models for the selected outcomes in Table A4 for males, specifically based on the TG2 sample, showing the estimated difference in log-odds (or means) of these outcomes compared to the “something else” subgroup. We see marginal evidence of differences between the “something else” subgroup and selected other subgroups for some of these outcomes (i.e. confidence intervals that only slightly cover zero), where the “something else” subgroup tends to have higher probabilities of the substance use outcomes (e.g., compared to bisexuals) after adjusting for the covariates.

**Table A8:** Estimated multivariable logistic regression models for selected outcomes for males, specific to group TG2 (with “something else” as a reference category).

|  | Past-year Cigarette Smoking | Past-year Other Drug Use | Household Size |
| --- | --- | --- | --- |
|  | n = 4,198 | n = 4,194 | n = 4,201 |
|  | Coefficient (95% CI) | Coefficient (95% CI) | Coefficient (95% CI) |
|  |  |  |  |
| **Sexual Identity** |  |  |  |
| Something Else | REF | REF | REF |
| Heterosexual | -0.13 (-0.87, 0.60) | -0.65 (-1.50, 0.20) | 0.16 (-0.07, 0.39) |
| Gay | 0.20 (-0.57, 0.98) | -0.39 (-1.46, 0.69) | -0.28 (-0.80, 0.23) |
| Bisexual | -0.92 (-1.86, 0.02) | -0.86 (-2.04, 0.32) | 0.16 (-0.28, 0.60) |

*P<0.05, **P<0.01, ***P<0.001

All models controlled for age, educational attainment, family income, race, and Hispanic ethnicity

Table A9 presents the same analysis for females, again showing evidence of differences in selected outcome measures for “something else” identifiers versus the other sexual identity subgroups after adjustment for covariates (e.g., a lower probability of past-year marijuana use, compared to bisexuals).

**Table A9:** Estimated multivariable logistic regression models for selected outcomes for females, specific to group TG2 (with “something else” as a reference category).

|  | Past-year Marijuana Use | Lifetime STD Status | Want a Child/Another Child in Future |
| --- | --- | --- | --- |
|  | n = 5,084 | n = 5,088 | n = 4,949 |
|  | Coefficient (95% CI) | Coefficient (95% CI) | Coefficient (95% CI) |
|  |  |  |  |
| **Sexual Identity** |  |  |  |
| Something Else | REF | REF | REF |
| Heterosexual | -0.78 (-1.23, -0.34)** | -0.53 (-1.21, 0.15) | 0.24 (-0.48, 0.95) |
| Lesbian | 0.14 (-0.58, 0.87) | -0.18 (-1.50, 1.14) | 0.20 (-0.60, 1.00) |
| Bisexual | 0.75 (0.23, 1.27)** | 0.16 (-0.64, 0.96) | 0.43 (-0.39, 1.25) |

*P<0.05, **P<0.01, ***P<0.001

All models controlled for age, educational attainment, family income, race, and Hispanic ethnicity
